# Thermally enhanced polyolefin composites: fundamentals, progress, challenges, and prospects

**DOI:** 10.1080/14686996.2020.1820306

**Published:** 2020-11-02

**Authors:** A.U. Chaudhry, Abdel Nasser Mabrouk, Ahmed Abdala

**Affiliations:** a Chemical Engineering Program, Texas A&M University at Qatar, Doha, Qatar; b Qatar Environment and Energy Research Institute, Hamad Bin Khalifa University, Doha, Qatar

**Keywords:** Polyolefin, thermal conductivity, composite interfaces, thermal transport mechanisms, thermal conductivity measurements, polyethylene, polypropylene, 103 Composites, 104 Carbon and related materials, 105 Low-Dimension (1D/2D) materials, 106 Metallic materials, 210 Thermoelectronics / Thermal transport / insulators, 302 Crystallization / Heat treatment / Crystal growth, 212 Surface and interfaces, 303 Mechanical / Physical processing, 700 Others

## Abstract

The low thermal conductivity of polymers is a barrier to their use in applications requiring high thermal conductivity such as electronic packaging, heat exchangers, and thermal management devices. Polyolefins represent about 55% of global thermoplastic production, and therefore improving their thermal conductivity is essential for many applications. This review analyzes the advances in enhancing the thermal conductivity of polyolefin composites. First, the mechanisms of thermal transport in polyolefin composites and the key parameters that govern conductive heat transfer through the interface between the matrix and the filler are discussed. Then, the advantage and limitations of the current methods for measuring thermal conductivity are analyzed. Moreover, the progress in predicting the thermal conductivity of polymer composites using modeling and simulation is discussed. Furthermore, polyolefin composites and nanocomposites with different thermally conductive fillers are reviewed and analyzed. Finally, the key challenges and future directions for developing thermally enhanced polyolefin composites are outlined.

## Graphical abstract

**Figure:**
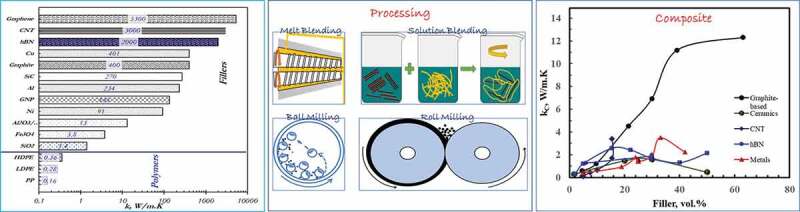

1.

## Introduction

**Figure 1.**
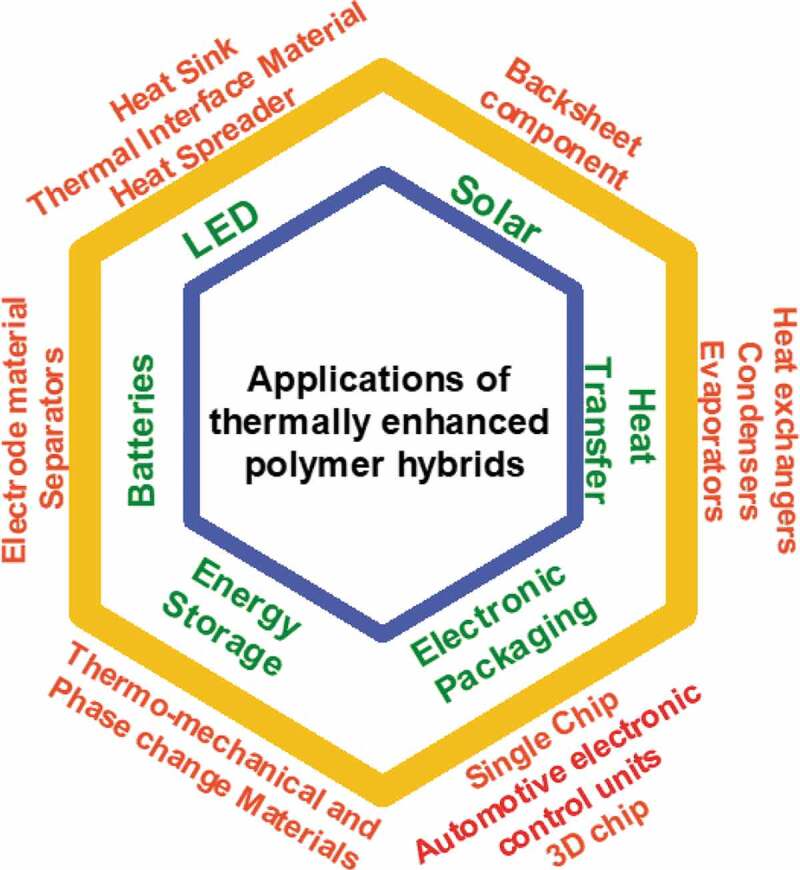
Potential applications of thermally enhanced polymers in current and emergent applications

Because of their low cost, ease of processing, the various functionalities, excellent corrosion, and chemical resistance, and lightweight, polymers have received considerable attention over the last few decades in emerging technologies (Figure 1) such as heat exchangers, waste energy recovery, heat dissipation applications, electronic packaging, solar, satellite devices, aerospace applications, renewable energy system, electronics, and Li–ion batteries [1–9]. However, the relatively low thermal conductivity of polymers (*κ_p_*) poses a challenge and constitutes a bottleneck towards commercial implementation. Therefore, improving the low *κ_p_* (0.1–0.5 W/m·K) is of great importance.

**Figure 2.**
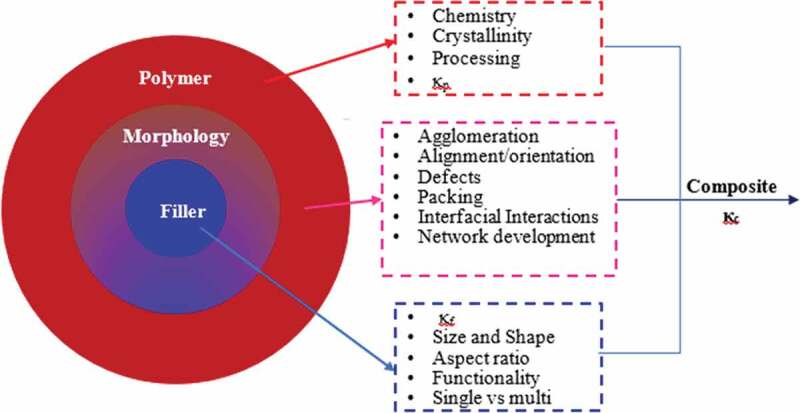
Design parameters for thermal conductivity of polymer composites

Many approaches are employed to enhance the poor *κ* of polymers; some of them rely on alignment of the polymer chains using mechanical stretching, electrospinning, and nanoscale templating [10] or fabricating polymer composites (PC) or nanocomposites (PNC) using highly conductive fillers. Polymers containing highly conductive micro/nano-particle fillers are desirable thermally conductive materials and have increased widespread interest. Along with enhanced *κ*, polymer filled with highly conductive filler also provide other benefits such as improved electrical conductivity (in some systems), magnetic permeability, and mechanical properties [11]. However, enhancement in *κ* of polymer composites (*κ_c_*) reported in the literature is relatively low compared to the predicted *κ_c_* based on the intrinsic filler conductivity (*κ_f_*) and filler loading. This lower measured *κ_c_* arises from the large interfacial thermal resistance (ITR) between the conductive filler and the surrounding polymer chains leading to phonon scattering and acting as rate-limiting in the thermal conductive pathway [12]. Moreover, *κ_c_* is affected by other multiple factors, including morphological properties of the composite, properties of the polymer, and the microstructure of the composite (Figure 2) that should be considered while designing polymer composite systems as will be discussed in forthcoming sections [9,13].

Among thermoplastics, polyethylene (PE) and polypropylene (PP) account for more than ~50% of the global polymer production due to their low cost and toxicity, and availability of a wide range of commercial grades in terms of PP forms with different chain structures, crystallinity, and density levels, and stereo configurations of varying densities: isotactic, syndiotactic, and atactic forms of PP. According to a 2020 report, polyolefins are the world’s fastest-growing polymer family with PE and PP accounting to nearly ~30% and ~20%, respectively, of total world polymer demand in 2018 [14]. The production of PE and PE represents a market size of valued 270.7 USD billion and ‘*is forecasted to grow at a compound annual growth rate of 6.2% from 2018 to 2026*’ [15]. Furthermore, they are the two largest thermoplastics by volume, which are fabricated into filaments, films, profiles, and moldings [16]. The main advantages of polyolefins, PE and PP in particular, over other polymers include the lightweight, low price, recyclability, easy and low-temperature processability, inertness, hydrophobicity, inertness and non-toxicity, excellent resistance to corrosive solvents, biocompatibility, rigidity, malleability, stiffness, low-temperature impact resistance, and high impermeability. These are the simplest and among the well-studied polymers having a wide range of commodity and engineering applications ranging from plastic bags to medical devices, including orthopedic implants, automobile parts, consumer goods, durable equipment, and industrial machinery [17–20]. Despite many benefits associated with neat polyolefins, polyolefin composites/nanocomposites emerged to meet the increased applications not satisfied by neat polyolefins. The recent advances in pristine polyolefin, polyolefin composite, and nanocomposite materials were comprehensively reviewed [19,21,22]. However, advances in the application of polymers in heat management areas such as circuit boards, heat exchangers, as replacement of metals or other materials, have also driven several recent studies on *κ* enhancement in polyolefin as well as other polymer composites. It was reported that *κ_p_* of a single PE nanofiber could reach 100 W/m·K, indicating the potential it can achieve after altering the molecular structure or the morphology [23]. *κ* enhancement of other polymers filled with fillers having high *κ_f_* has been investigated extensively. For instance, the study of *κ_c_* of poly(vinyl butyral), poly(ethylene vinyl alcohol), poly(methyl methacrylate), and polystyrene-based nanocomposites filled with 24 wt.% boron nitride nanotubes exhibited *κ_c_* of 1.80, 2.50, 3.16, and 3.61 W/m·K, respectively [12,24]. Similarly, the addition of 15 and 40 wt.% graphite to thermoplastic polyetherimide improved *κ_p_* from 0.07 to *κ_c_* of 0.87 and 1.73 W/m·K, respectively. Moreover, incorporation of 50 wt.% glass fiber into thermoset polyetherimide increased *κ* to 0.41 W/m·K. In their reports, *κ* of increased from *κ_p_* polyethylene terephthalate, i.e., 0.15 W/m·K, increased to *κ_c_* of 0.31, 0.71 and 0.72 W/m·K using 45% glass fiber, 30% graphite fiber, and 40 wt.% PAN carbon fiber, respectively [11].

Polyolefin composites have several advantages over other polymer composites, including the low material costs, ease of fabrication and good manufacturability, excellent chemical and corrosion stability, and the broad range of mechanical properties. Owing to their simple backbone structure, polyolefin chains, especially PE composites, exhibit a large change in *κ_c_* upon alignment. Therefore, using filler with high *κ*_f_ combined with the simultaneous alignment of PE chains and the embedded filler, *κ_c_* can be significantly enhanced. For instance, *κ_c_* of PE composite containing 10 wt.% graphene nanoplatelets has *reached* 5.9 W/m·K after stretching the composite, indicating the great potential to fabricate polyolefin composites with high *κ_c_* [25].

The current interest in thermally enhanced polyolefin to meet the requirements of many emerging applications focuses on an excellent combination of thermal and mechanical properties. Several reviews discuss the progress in improving *κ* of polymers via controlling/altering the polymer morphology [22] and fabrication of composites and nanocomposites with conductive fillers [10–12,26,27]. However, to the best of our knowledge, no review focuses on enhancing *κ* of polyolefin via the fabrication of composites with conductive fillers and nanofiller. This comprehensive review is dedicated to the progress in improving *κ_c_* of polyolefin composites. Polyolefin are a unique class of polymers, not only because of their vast production scale and low cost but also because they are the most challenging polymer for the fabrication of composites and nanocomposites due to their nonpolar nature that leads to poor dispersion of the filler and weak interface between the polymer and the filler. This weak interface promotes phonon scattering that limits the enhancement in *κ_c_*. A review dedicated to this class of polymers provides an in-depth analysis of the key aspects that govern the enhancement of *κ* in terms of processing, filler type, and loading. Moreover, other essential elements such as heat transfer mechanism in polymer composites, modeling, and simulation of thermal conductivity in polymers and polymer composites, and thermal conductivity measurement techniques are also discussed. Therefore, this comprehensive review will contribute to accelerating the development of commercially viable thermally conductive polyolefins.

This review analyzes the advances on enhancing the *κ* of polyolefin composites. First, the microscopic origin of *κ*, the mechanisms of thermal transport in polymers and the key parameters that govern conductive heat transfer through polymers and polymer composites are briefly discussed. Next, transient and steady-state techniques for measuring *κ* are analyzed. Then, the microscopic thermal transport mechanisms in polymer composites (PC) are discussed. Theoretical progress in predicting *κ* of the polymer composites containing micro- and nano-conductive fillers are presented.

Furthermore, a comprehensive analysis of the enhancement in *κ* of polyolefins reinforced with carbon-based, ceramic, and metallic micro- and nano-fillers are scrutinized. The impact of processing method and condition, the filler conductivity, size, shape, and aspect ratio, combining hybrid fillers, controlling filler orientation, and controlling the composite microstructure on *k*_c_ of the polyolefin composites is discussed. Finally, the current challenges and future research directions are presented.

## 2. Thermal conductivity

**Figure 3.**
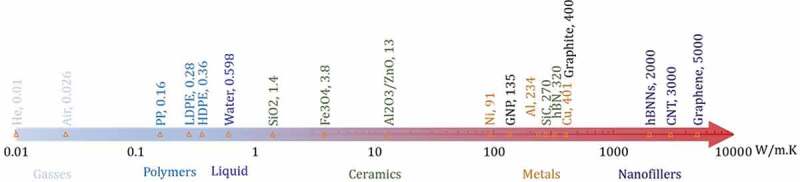
Thermal conductivity of different materials

Thermal conductivity, denoted as Greek letter *κ* or small letter *k*, is a material-intensive property that measures the material ability to transport heat molecularly (conduction). Macroscopically, *κ* is the constant of proportionality in Fourier’s Law for isotropic, homogeneous material:

(1) $$J=K\frac{\Delta T}{\Delta x}$$

where J is the heat flux (W/m^2^), *κ* is the thermal conductivity of the material (W/m·K); ΔT is the differential temperature across the sample (°C); Δx is the differential thickness or the conduction path length (m). Figure 3 presents the variation in *κ* for common types of materials. *κ* generally varies from 0.01 W/m·K for gases such as helium, 0.15–0.5 W/m·K for polymers, reaches 400 W/m·K for highly conductive metals such as copper and also graphite, and up to ~5000 W/m·K for single-layer graphene.

**Figure 4.**
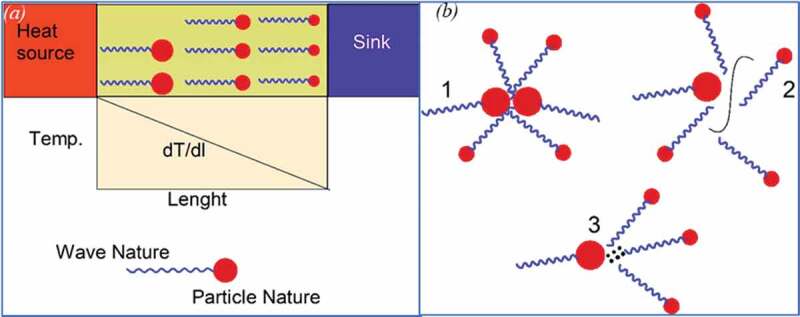
Graphical representation of (a) a phonon and change in phonon energy while traveling from the heat source to the sink (larger head represent the higher energy phonons and vice versa) and (b) different modes of phonon scattering (1) phonon-phonon, (2) phonon-boundary, (3) phonon-impurity [22,27]

From a microscopic viewpoint, *κ* of solids is attributed to the transport of energy carriers such as phonons, electrons, or photons. Generally, in crystalline solids, atoms continuously experience coupled and coordinated vibrations with high frequency and comparatively small amplitudes (shorter wavelengths), which can be assumed as elastic waves. On the other hand, phonons transport is the predominant mechanism for thermal conduction in polymers and PC, and it can be described as quantized modes of vibration. Phonons are analogs to the photons having wavelike and particle nature as schematically presented in Figure 4a that depicts the evolution of temperature gradient across the material thickness. The heat conduction in solids takes place by high-energy phonons or energy-carrying packets generated by the lattice vibrations. The thermal phonons start their journey from the hot source and gradually lose their energy as moving towards the heat sink, creating a temperature gradient across the conducting medium. Moreover, Figure 4b also indicates the origin of thermal resistance, which arises from different modes of phonon scattering owing to imperfections in the solids [22,27].

### 2.1 Thermal conductivity measurements

Precise measurements of thermal transport properties of polymers and PC are vital in thermal management applications. Numerous standards, methodologies, and procedures exist to measure the thermal properties of polymers correctly. Recently, Palacios et al. [28] have published a comprehensive review of the measurement methods, equipment, and sample preparation for characterizing thermal energy storage materials. The thermal conductivity of solid materials is greatly affected by the measurement conditions, as well as the technique used. The precision of the measurement techniques has been considered as possible contributor to the discrepancy in the reported values of *κ_p_* and *κ_c_*.

**Figure 5.**
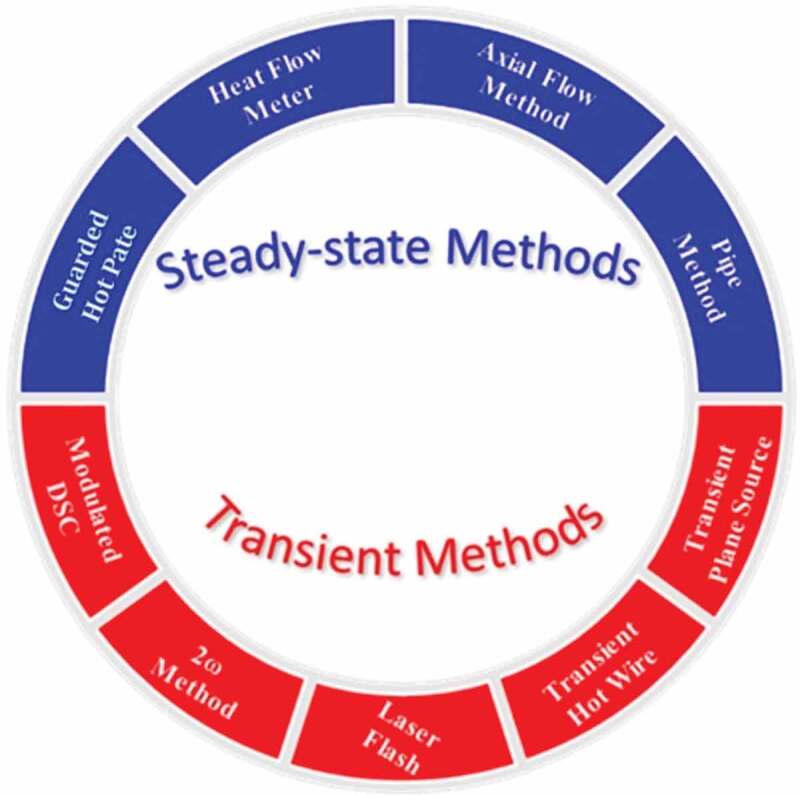
Steady-state and transient thermal conductivity techniques [28]

The methods for measurement of thermal transport properties (diffusivity and conductivity) are classified as either steady-state methods (guarded hot plate and heat flow meter) or transient methods (transient plane source, transient hot wire, laser flash, modulated differential scanning calorimetry (DSC), 3 ω, thermocouple) as schematically depicted in Figure 5 [28]. Steady-state methods directly measure *κ* and the interfacial thermal conductance after the system has attained stability, i.e., under a steady-state heat flow. On the other hand, transient methods measure thermal diffusivity while heating. The steady-state techniques are suitable for the *κ*, composite, and anisotropic materials, and required comparatively large samples [29]. These methods have disadvantages as they suffer from parasitic heat losses and contact resistance [28]. Transient methods overcome these drawbacks by applying either periodic or transient heat input and measuring the change in sample temperature versus time. Transient methods determine the thermal diffusivity (α) during the heating process, and *κ* can be estimated using the relation:

(2) $$\alpha =\rho C_{p}\kappa$$

where *C_p_* is the specific heat capacity, and ρ is the mass density. Other advantages of the transient methods are the short measurement time, small sample requirement, measurements over a wide temperature range, and the ability to measure simultaneously α and κ. A summary of different *κ* measurement methods is presented in Table 1. The selection of the technique to use depends primarily on the measurement temperature range, the material type, the inherited *κ*, measurement time, accuracy, and sample geometry [30].

**Table 1.** Thermal conductivity measurement techniques. Reproduced from Ref [44]. Copyrights 2016 Yuksel, Licensee IntechOpen. Copyright (2017), and Ref [28] with permission from Elsevier”

Technique		*κ* range W/m·K	Working principle	Materials type	Advantages/ *Disadvantages*
Steady-State	*Guarded hot plate*	<0.8		Solid, opaque, insulators	high precision/*large sample size, limited to low κ materials*
	*Axial flow*	0.2–200		Metals	Wide temperature range/*prolonged measurement time*
	*Heat flow meter*	<10		Polymers, ceramics, some metals	Simple setup and operation/*High uncertainty*
	*Pipe*	0.02–200		Ceramics	Wide temperature range/*sample preparation, prolonged measurement time*
Transient	*Laser flash*	>0.01		Solids and liquids, metals, not for insulating materials	Wide temperature range, small sample, very fast, high precision, high temperature/*high cost*
	*Transient hot wire*	<25		Liquids, low *κ* materials	Wide temperature range, quick, precise/low *κ* materials
	*Transient plan source*	0.005 − 1800		liquids, aerogels and solids	Solid, liquid and powder state/*less accurate, large sample size*
	*3 omega (3 ω)*	0.20–20		Thin films (> 100 nm) and nanowires liquids, other non-electrically conductive, porous materials	Simple sample preparation, short measuring time, small sample/*complex evaluation*
	*Modulated DSC*	0.10 − 1.0		Solid materials, thin samples, polymers, glass,ceramic,	Wide temperature range, very small sample/c*omplex evaluation*

Among the steady-state techniques, the *guarded hot plate (HGP)* method is used for low *κ* materials in a broad temperature range, i.e., 80–1500 K. It provides high accuracy (<2% error) and it requires long measurement time and large sample size. In HGP method, the sample is placed between a cold plate and a hot plate attached to heaters/cooler and thermally insulated. The heat passes through the specimen, and after the system reaches a steady-state, the temperature of both sides of the specimen is measured. *κ* is calculated based on the measured, steady-state heat flux, the temperature gradient across the sample, and the thickness and surface area of the specimen [31].

*The axial flow method* is a longitudinal method that measures a wide range of conductivities at low temperatures by creating temperature difference across a sample placed between two suitable references of known *κ* attached to a heater and a heat sink [32]. *The heat flow meter method* is similar to the guarded hot plate method, but the device has a heat flux sensor instead of the main heater. It is simple in construction and operation and able to operate in temperature ranges of −73 ‒ 473 K, 90 ‒ 1300 K, or 298 ‒ 2600 K for normal, axial, and radial heat flow, respectively [33]. *The pipe method* is another radial heat flow method that operates at a wide range of temperatures (293–2700 K) and *κ* between 0.02 and 2 W/m·K. It follows the same principle of temperature gradient using a cylindrical sample containing a core heater and surrounded by a heat sink [30,34].

Among the transient methods, the *laser flash method* is one of the most common and versatile techniques. It is fast, easy to operate, non-contact, and non-destructive technique that provides very high accuracy. In this method, a pulse supplied by a laser beam source heats the surface of a disk, square, or rectangular sample, and an infrared detector monitors the temperature change on the opposite sides of the sample. The thermal diffusivity is measured as:

(3) $$\alpha =0.1388\;\frac{d^{2}}{t_{1/2}}$$

where α is the thermal diffusivity (cm^2^/s), d is the sample thickness (cm), t_1/2_ is the time (s) at which 50% of the maximum temperature increase is reached [28].

*The transient plane source* method, also known as the hot disk method, is similar to the pipe method. It uses a hot wire embedded in the test sample and functions as a temperature sensor. A known small and constant electrical current pulse heats the sample using a heating element placed between two specimens of the test material. The temperature change is recorded as a function of time, and *κ* is calculated as follows [35–39]:

(4) $$\kappa =\frac{P_{0}}{r\Delta T\left(\tau \right)\pi {\;}^{\frac{3}{2}}}D_{s}\left(\tau \right)$$

where P_0_ is the power input to the sensor; r is the outer ring heater radius; τ is non-dimensional time; ΔT(τ) is the temperature rise on the sample surface; D_s_(τ) is the shape function of τ [40].

*The transient hot-wire method* is commonly used for measuring *κ* of liquids. In this method, a heated wire acts as a heat source, output heater, and a temperature detector. The heat spreads out-radial into the sample, and the slope of the temperature rise is proportional to the time-lapse. According to the transient hot-wire method, *κ* is calculated using the following equation:

(5) $$\kappa =\frac{q}{\Delta T4\pi }\ln\frac{t_{2}}{t_{1}}$$

where q is heating power per unit length (W/m), and T is wire temperature at time t (s) [41,42].

*The conventional DSC method* is commonly used to determine the thermal properties of materials. Modified DSC or Temperature *modulated DSC method* can also be used to determine *κ* of insulating materials using linear heating and a superimposed oscillatory temperature program to create cyclic heating of the sample. The modulated DSC method uses the same setup and sample size as conventional DSC. For low *κ* and cylindrical samples, *κ* can be calculated using Equation 6:

(6) $$\kappa =\frac{8LC^{2}}{C_{p}mPd^{2}}$$

where C is the apparent heat capacity (mJ/K); L is sample thickness (mm); m is sample mass (mg); d is sample diameter (mm); P is the modulation period (s) [28,43].

*3 ω* is another transient technique similar to the hot wire method, but it works in a specific frequency domain. *3 ω* method is used to measure *κ* of liquids, thin films (>100 nm), and nanowires. During the measurement of *κ*, an AC current with frequency ω is passed through the wire, which acts as a heater and thermometer, and the response is measured as temperature oscillation indirect determined from the 3 ω voltage. Because the current is driven at frequency ω and the resistance changes at frequency 2 ω, a voltage results at 3 ω. Compared to other methods, *3 ω* requires shorter equilibration times and minimizes radiation losses [44].

## 3. Theoretical aspects of thermal conductivity in polymer composites

### 3.1 Mechanism of thermal transport through polymer composites

A common approach to enhance *κ_p_* is through the fabrication of a composite with thermally conductive fillers that have much higher *κ* compared to the polymer matrix. The most critical factors that dictate *κ_c_* are the intrinsic polymer conductivity (*κ_p_)*, the filler conductivity (*κ_f_)*, the filler volume fraction (ϕ_f_), the filler size and shape, the distribution and dispersion of the filler into the matrix, and the strength of the filler–matrix interface. Composites containing fillers with a high aspect ratio (*a*) generally have low percolation threshold and therefore yield higher *κ_c_* compared to fillers with lower *a* at the same ϕ [13,45]. Traditionally, higher filler loading (>30 vol.%) is necessary to achieve high *κ_c_*. Moreover, fillers with platelet shape are more effective than spherical or cylindrical fillers as the plate-like structure reduces the thermal contact resistance by overlapping large contact area and permitting much closer contact between adjacent platelets. The interfacial thermal contact resistance can also be reduced by decreasing the interface (edge) and increasing the size of the conductive particles or the number of clusters [46]. In addition to the intrinsic filler properties, *κ*_c_ also depends on other factors such as the filler loading, shape and size, and the filler-matrix interfacial adhesion. In General, *κ*_c_ has a non-linear dependence on the filler loading and higher filler loading is required to achieve filler-to-filler connections necessary to improve *κ_c_* [47]. Moreover, the filler shape also has a substantial effect on *κ*_c_ as fillers with low *a* yield lower *κ*_c_ compared to fillers with high *a* (fibers, tubes) at the same loading.

**Figure 6.**
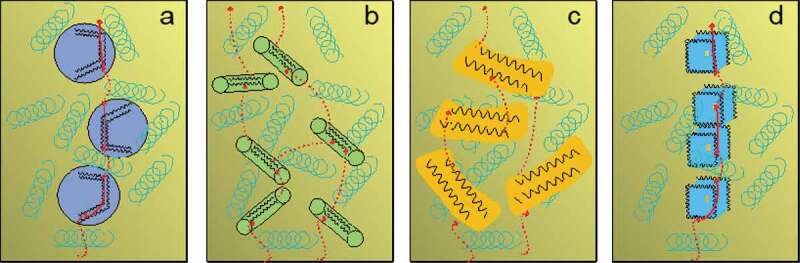
Modes of thermal transport in polymer composites systems incorporated with different filler dimensions (a) 0D fillers, (b) 1D fillers, (c) 2D fillers, (d) 3D fillers [54]

Based on their dimensionality, the thermally conductive fillers can be categorized into 0D, 1D, 2D, and 3D fillers. Generally, 0D fillers are considered as point-like particles including sphere or cluster-like structure, (2) 1D fillers include nanofibers, rods, wires, etc. (3) 2D fillers are sheets or layer type structures, and (4) 3D fillers include 3D network-like fillers (Figure 6) [48,49]. For each filler type, the primary mechanism of *κ_c_* is related to the formation of a conductive network in the PNC and the reduction of the filler-filler ITR. In general, 0D fillers require higher loading to obtain high *κ*_c_ compared to 1D and 2D fillers. Higher loadings do not only increase the composite cost but also increase the composite melt viscosity making the composite processability poor.

On the other hand, 1D and 2D fillers with high *a* can significantly improve *κ*_c_ as they generate prolonged heat conductive pathways. Moreover, *κ*_c_ of composites with aligned 1D and 2D fillers is anisotropic with high value along the filler alignment direction. Examples of thermally conductive 1D fillers are carbon nanotubes, carbon fibers, Si_3_N_4_ nanowires, boron nitride nanotubes, silver nanowires, and copper nanowires [50–52]. 2D conductive fillers with high *a* such as thin platelets perform well when incorporated in the polymer matrix. Examples of conductive fillers with plate-like morphology are boron nitride, graphene, Al_2_O_3_, TiB_2,_ and SiC [53].

**Figure 7.**
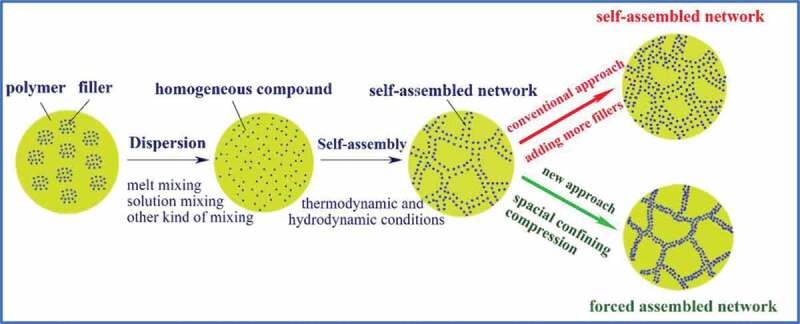
Schematics of self-assembled and forced assembled thermal conductive networks prepared by traditional compounding and spatial confining forced network assembly methods..‘Reprinted from Ref [57]. Copyright (2018), with permission from Elsevier’

Composites made with 3D-layered fillers such as hBN and graphite exhibits anisotropic *κ_c_* with high value in the in-plane direction compared to the orthogonal direction. 3D network-like fillers can overcome the drawbacks of filler aggregation and reduces ITR of the matrix-filler and filler-filler and thus provide a more stable 3D thermal transport network that significantly enhances *κ*_c_ [10]. The spatial confining forced network assembly (SCFNA) technique was adopted to composites containing 3D network structure that high *κ*_c_ as shown in Figure 7 [54–56].

Furthermore, the filler size greatly influences *κ_c_* as small size fillers result in larger interfacial area and more pronounced phonon scattering. In contrast, large fillers are more effective in creating a percolated network, which reduces the thermal interfacial resistance. Nonetheless, some reports claim *κ_c_* is independent of the particle size as nano-size fillers resulted in similar *κ*_c_ enhancement as microsize fillers [58]. Also, the dependence of *κ_c_* on network formation by conductive fillers is well established regardless of the filler size and thermal resistance between the filler and the matrix [39,58–60]. Furthermore, *κ_c_* can also be improved by other techniques such as using hybrid fillers with different sizes, shapes, and types as well as surface treatment of the filler [61–63]. Moreover, the composite microstructure that develops during processing as dictated by the processing conditions and the ability/tendency of the filler to orientate, agglomerate, and form network also affects *κ_c_*. During processing, the filler particles can be oriented using externally applied fields and shear or extensional flow. Similarly, the formation of a filler network can be achieved by self-assembly of the filler particles in the polymer matrix, molding of the mixture of the filler and polymer powders, in situ polymerization or double percolation. Agglomeration of the filler is sometimes necessary to achieve high and isotropic *κ_c_* [64–70]. Filler dispersion in the host matrix is significantly impacted by the processing method. *κ_c_* for composites with the same filler loading can vary considerably based on the processing method following the order [71,72]:

Powder mixing > Solution blending > Roll − mill blending > Melt blending

The thermal conductance mechanism in PC also depends on the effective inter-filler network. Generally, at low concentration and in the absence of an inter-filler network, the composite exhibit low *κ_c_* due to the high thermal resistance and vice versa. Moreover, despite the development of filler network, *κ_c_* might not show much improvement, possibly due to the high inter-filler thermal resistance arises caused by the filler geometric, shape, arrangement, and connections.

### 3.2 Modeling of thermal conductivity of polymer composites

Modeling provides a powerful tool to predict the effect of different factors on *κ_c_*. Therefore, both theoretical modeling and molecular simulations can be used to guide the design of composite materials. Theoretical modeling, both classical and novel model for composite materials, describes the heat transfer mechanism in composites. Molecular simulations methods describe the characteristics of the microstructure at different length scales and provide information on the interface resistance and *κ_c_* of bulk composites at different length scales. Modeling of pure polymers is relatively straightforward compared to modeling composite systems due to the evolution of complex morphology during the composite processing, i.e., poor dispersion of fillers, network formation, and interfacial resistance. Numerical models used to predict *κ_c_* are qualitative or semi-quantitative. In the next section, we briefly discuss this theoretical modeling of *κ_c_*, and we refer the reader to general review on modeling *κ*_c_ of PCs [8]

#### 3.2.1 Theoretical and numerical modeling

As mentioned earlier, theoretical models that predict *κ_c_* are either based on classical theoretical models that lead to the effective medium approximation (EMA) or micromechanics (MM) method. Before discussing EMA, two main simple approaches are adopted as a first approximation to predict *κ_c_*, i.e., upper bound (parallel model, linear mixing rule) and the lower bound (series model, inverse mixing rule). In the basic parallel model, the overall conductivity is independent of each phase, i.e., the filler and matrix, and the heat flux is the weighted sum of the heat flux through the domain of each phase while the temperature gradient is uniform. The parallel model also assumes the particles are in complete contact, forming a percolating network. According to the parallel model, *κ_c_* is a function of *κ_p,_ κ_f,_ ϕ_p, and_ ϕ_f_* as follows:

(7) $$\kappa_{c}=\kappa_{p}\phi_{p}+\kappa_{m}\phi_{m}$$

where the subscripts c, p, and m refer to the composite, the polymer, and the filler, respectively. On the contrary, the basic series model assumes that the temperature gradient is the weighted sum of the temperature gradient through the domain of each phase, and the heat flux is uniform across all phases. It also assumes the filler particles are entirely out of contact with each other, and it is not possible to reach percolation. According to the series model, *κ* is calculated as follows [13,73]:

(8) $$\kappa_{c}=\frac{1}{\left(\kappa_{p}+\Phi_{p}\right)+\left(\kappa_{m}+\Phi_{m}\right)}$$

Between these two models, the inverse mixing rule (series model) is more common, and its prediction provides a better description of the experimental data. The series model has also served as the basis of many models built on complex weighted averages of *κ*_c_, *κ*_p_, $\Phi_{p}$ and $\Phi_{m}$. These complex models frequently consider semi-theoretical fitting parameters and depend on EMA or effective medium theories (EMT). Based on the EMA approach, many standard models were proposed, such as the Maxwell model, Maxwell–Grant model, Bruggeman–Hanai model, Bruggeman–Landauer model, Hasselman–Johnson model, Rayleigh models, Frick’s model, Nan’s model, Lewis–Nielsen model, Percolation model, and Every model [74,75]. Maxwell model considers spherical non-interacting particles embedded in a continuous matrix where effective *κ* of the composite *κ*_eff_ is given by:

(9) $$\kappa_{eff}=\kappa_{m}\left[1+\frac{3\Phi_{f}}{\frac{\kappa_{f}+2\kappa_{m}}{\kappa_{f}-\kappa_{m}}-\Phi_{f}}\right]$$

The validity of Maxwell’s model was limited to filler volume fraction <25, % which has led the development of other modifications for particle shape and different phases of filler particles [76,77]. Similarly, Rayleigh introduced another model based on the consideration of thermal interaction between particles. This model considers the fillers as cubically arranged spherical shape inclusions in the matrix and can be used for higher volume fractions of filler. According to the Rayleigh model, *κ_eff_* can be calculated as [78]:

(10) $$\kappa_{eff}=\kappa_{m}\left[1+\frac{3\Phi_{f}}{\left(\frac{\kappa_{f}+2\kappa_{m}}{\kappa_{f}-\kappa_{m}}\right)-\Phi_{f}+1.569\left(\frac{\kappa_{f}-\kappa_{m}}{3\kappa_{f}-4\kappa_{m}}\right)\Phi_{f}^{\frac{10}{3}}+\;\ldots \;.}\right]$$

Using Maxwell and Rayleigh models, the Hasselman–Johnson novelty model considered the fillers volume fraction, interfacial gaps/thermal resistance, and particle size to develop the k_eff_ formula. According to Hasselman–Johnson model equation of *κ_eff_* for spherical, cylindrical, and flat plate geometry are given as:

(11) $$\kappa_{eff}=\kappa_{m}\left[\frac{2\left(\frac{\kappa_{f}}{\kappa_{m}}-\frac{\kappa_{f}}{ah_{c}}\right)\Phi_{f}+\frac{\kappa_{f}}{\kappa_{m}}+\frac{2\kappa_{f}}{ah_{c}}+2}{\left(1-\frac{\kappa_{f}}{\kappa_{m}}+\frac{\kappa_{f}}{ah_{c}}\right)\Phi_{f}+\frac{\kappa_{f}}{\kappa_{m}}+\frac{2\kappa_{f}}{ah_{c}}+2}\right]\;\;\;\;\;Spherical$$

(12) $$\kappa_{eff}=\kappa_{m}\left[\frac{\left(\frac{\kappa_{f}}{\kappa_{m}}-\frac{\kappa_{f}}{ah_{c}}-1\right)\Phi_{f}+\left(1+\frac{\kappa_{f}}{\kappa_{m}}+\frac{2\kappa_{f}}{ah_{c}}\right)}{\left(1-\frac{\kappa_{f}}{\kappa_{m}}+\frac{\kappa_{f}}{ah_{c}}\right)\Phi_{f}+\left(1-\frac{\kappa_{f}}{\kappa_{m}}+\frac{\kappa_{f}}{ah_{c}}\right)}\right]\;\;\;\;\;\;\;Cylindrical$$

(13) $$\kappa_{eff}=\left[\frac{\kappa_{f}}{\left(1-\frac{\kappa_{f}}{\kappa_{m}}+\frac{2\kappa_{f}}{ah_{c}}\right)\Phi_{f}+\left(\frac{\kappa_{f}}{ah_{c}}\right)}\right]\;\;\;\;\;Flat\;plate$$

where *a* and *h*_c_ represent the particle radius and the boundary conductivity, respectively [79,80]. Furthermore, Bruggeman used differential equations to calculate infinitesimal changes in the incrementally constructed composite system. This approach is usually called differential effective medium theory/scheme (DEM) and can be used for different systems and high filler volume fractions,$\;\Phi_{f}$. Using Bruggeman approach, many researchers obtained *κ_eff_* for different systems given as:

(14) $${\left(1-\Phi_{f}\right)}^{3}={\left(\frac{\kappa_{m}}{\kappa_{eff}}\right)}^{\frac{\left(1+2\alpha \right)}{\left(1-\alpha \right)}}{\left[\frac{\kappa_{eff}-\kappa_{f}\left(1-\alpha \right)}{\kappa_{m}-\kappa_{f}\left(1-\alpha \right)}\right]}^{\frac{3}{\left(1-\alpha \right)}}\;\;\;\;Particulate$$

(15) $$\frac{\kappa_{f}}{\kappa_{m}}=\frac{1}{{\left(1-\Phi \right)}^{3\left(1-\alpha \right)/\left(1+2\alpha \right)}}\;\;\;\;\;\;\;ZnS-Diamond$$

(16) $$\left(1-\Phi_{f}\right)={\left(\frac{k_{m}}{k_{eff}}\right)}^{\frac{1}{3}}\left[\frac{\kappa_{eff}\kappa_{f}Ref{f_{1}}_{int}}{\kappa_{m}\kappa_{f}R{m_{1}}_{int}}\right]\;\;\;\;Composite$$

where α is a dimensionless parameter depends on ITR (R_int_) between filler and matrix and α = *a*_k_/*a*, where *a* is particle size, *a*_k_ is Kapitza radius, *a*_k_ = R_int_k_m_ [81,82]. Lewis–Nielsen model was another simple and popular model reported in the literature for moderate $\Phi_{f}$(<40%). The benefits of this model were its applicability for a broad range of particle shapes and arrangements. The *κ_eff_* of a composite according to this model is given as

(17) $$\kappa_{eff}=\frac{1+AB\Phi_{f}}{1-B\psi \Phi_{f}}$$

(18) $$B=\left(\frac{\left(\frac{\kappa_{f}}{\kappa_{m}}\right)-1}{\left(\frac{\kappa_{f}}{\kappa_{m}}\right)+A}\right)$$

(19) $$\psi =1+\left(\frac{1-\Phi_{m}}{\Phi_{m}^{2}}\right)$$

where $\phi_{m}\;$and A are the maximum filler volume fraction and shape coefficient for the filler particles, respectively [39,75,83,84].

Micromechanics models are useful for the rapid evaluation of *κ_c_* based on the composition and properties of the filler and the polymer. Still, their applicability is often limited to specific composite systems like homogenous matrices with monodisperse spherical or perfectly aligned ellipsoidal fillers. The primary micromechanics methods use variational principle (VP) and the mean-field approximation (MFA) to calculate *κ_eff_*. The VP includes Hashin–Shtrikman bounding while MFA includes Mori–Tanaka model and Benveniste’s model. Hashin–Shtrikman bounds provide the tightest possible range of variation for the property under study, knowing only volume fraction and macroscopic anisotropy. Hashin–Shtrikman bounds have been improved and modified by many authors [85,86]. The Mori–Tanaka model evaluates the *κ_eff_* in an isotropic matrix containing inhomogeneities distributive in the matrix under the condition that the temperature gradient and heat flux are uniform on the boundary [87,88]. Using MM analysis, Benveniste obtained expressions for composites with ITR, which evaluates the *κ_eff_* for multiphase systems by determining the average flux in each constituent [75,89].

The classical theoretical models were modified to develop novel models to analyze further the factors affecting *κ_eff_*. For instance, to consider particles of complex shape, Shahil and Balandin [90] modified Nan’s model by treating the layer as a multilayer graphene of different thicknesses. Accordingly, *κ_eff_* is given by:

(20) $$\kappa_{eff}=\kappa_{p}\frac{3\kappa_{m}-2f\left(\kappa_{p}-\kappa_{m}\right)}{\left(3-f\right)\kappa_{p}+\kappa_{m}f+\frac{R_{1}\kappa_{p}\kappa_{m}\;f}{nh_{m}}}$$

where n is the number of layers in the multilayer graphene. Similarly, to consider the folded and wrinkled nature of particles, Chu et al. [91] introduced the flatness ratio (F) for such particles. The *κ_eff_* of the composites on account of Nan’s model is given by:

(21) $$\frac{\kappa_{eff}}{\kappa_{m}}=\frac{3+\frac{2F^{2}f}{\kappa_{m}\left(\frac{2R_{1}}{Lh_{filler}}+13.4\sqrt{h}\right)}}{\left(3-Ff\right)}$$

Yu et al. [92] modified the Every’s model for hybrid particles introduced a synergistic term, s, for hybrid particles. The *κ_eff_* of composites based on Every’s model is given by:

(22) $$\frac{\kappa_{eff}}{\kappa_{m}}=\frac{S}{{\left(1-f\right)}^{3\left(1-\frac{\kappa_{m}R_{1}}{r}\right)/\left(1+\frac{2\kappa_{m}R_{1}}{r}\right)}}$$

**Figure 8.**
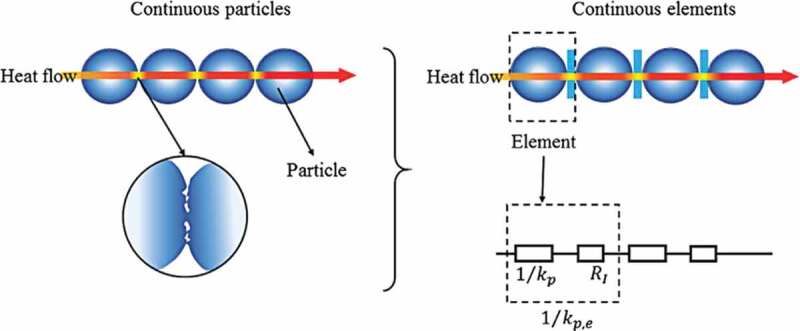
The schematic illustration of the connection mechanism of particles; the real particle contact on the left and the equivalent particles contact on the right. ‘Reprinted from Ref [8]. Copyright (2018), with permission from Elsevier’

Xu et al. [93] accounted for the effect of microstructures on *κ_eff_* by modifying the Maxwell–Garnett model using a linear combination of the mesoscale control volume. They considered the particle connection mechanism by adding the resistance between the continuous phase of particles (Figure 8) [8,93]. Based on the modified Maxwell–Garnett model, *κ_eff_* can be calculated from the relation

(23) $$\frac{\kappa_{eff}-\kappa_{m}}{2\kappa_{eff}+\kappa_{m}}\left(1-f\right)+\frac{\kappa_{eff}-\kappa_{p,e}}{2\kappa_{eff}+\kappa_{p,e}}=0$$

To study the effect of volume fraction on *κ_eff_, the* percolation model was modified to include the impact of the percolation phenomenon [94]. Therefore, *κ_eff_* of the composite is given by:

(24) $$\frac{\kappa_{eff}}{\kappa_{m}}=\frac{2/3{\left[f-f_{c}\left(p\right)\right]}^{\alpha }}{H\left(p\right)+1/\left(\frac{\kappa_{x}^{c}}{\kappa_{m}}-1\right)}+1$$

where *f*_c_(p) is approximately equal to 1/p and a is a fitting parameter.

All the theoretical models investigated above are appropriate for composite containing single filler only. However, incorporation of second or third filler usually gives a synergistic effect on the *κ_c_*. Hence, Agrawal and Satapathy [95] modeled the heat transfer mechanism in polymer composites containing multi-filler and the correlation calculating *κ_eff_* in terms of the volume fractions of individual filler *ϕ_i_* and their respective *κ_i_* as well as *κ_p_* is given by equation [95]:

(25) $$\frac{2}{\kappa_{eff}}\;=\underset{i}{\sum }\left[\frac{1}{\kappa_{p}}-\frac{1}{\kappa_{p}}{\left(\frac{12\phi_{i}}{\pi }\right)}^{\frac{1}{3}}+\frac{2}{\kappa_{p}{\left(\frac{2\pi }{3\phi_{i}}\right)}^{\frac{1}{3}}+{\left(\frac{4\phi_{i}}{9\pi }\right)}^{\frac{1}{3}}\pi \left(\kappa_{i}-\kappa_{p}\right)}\right]$$

Zhou et al. [96] modified the basic and straightforward model given in Equation 7 and calculated the *κ_eff_* of composites incorporated with high loadings, which is calculated using:

(26) $$\kappa_{eff}=\kappa_{o}\frac{\rho_{f}}{\rho_{o}}+\kappa_{r}\frac{\rho \left(1-f\right)}{\rho_{r}}$$

where *ρ* is the composite density, *ρ*_o_ is the density of oriented filler, and *ρ*_r_ is the density of random filler, which could be measured from completely random samples and *f* the Lotgering factors (orientation factor) which was calculated from x-ray diffraction analysis [96].

#### 3.2.2 Simulation of thermal conductivity in polymer composites

**Figure 9.**
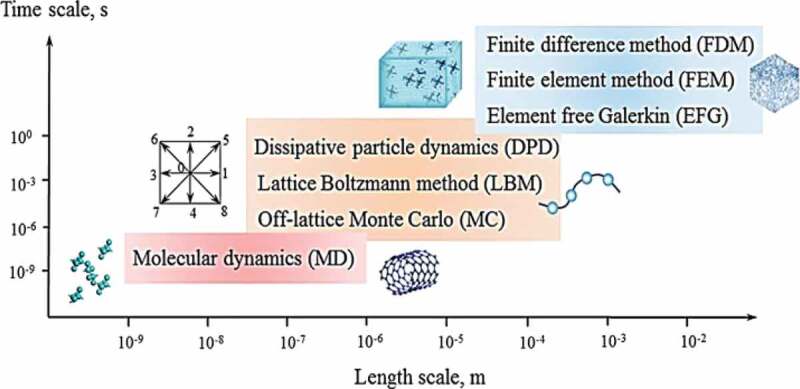
Time scale versus length scale of different simulation methods. ‘Reprinted from Ref [8]. Copyright (2018), with permission from Elsevier’

Predicting the composite conductivity using molecular simulation is attractive because of many reasons, such as these methods deeply analyze the *κ* and its correlations by considering realistic composite morphology and possible interfacial resistance. However, these methods are time and power-consuming and are frequently used as complementary tools to verify and test predictions and results of constitutive models [13,75]. Simulations methods are used to develop the model for interface resistance and bulk composites at different length scales, as shown in Figure 9.

Thermal conductivities of bulk composites were also simulated at different length scales. Table 2 summarizes the simulation models based on their length scale.

**Table 2.** Simulation models of thermal conductivity in bulk composites [8]

Length scale	Simulation method
Microscopic	Equilibrium molecular dynamics Non-equilibrium molecular dynamics Reverse nonequilibrium molecular dynamics
Mesoscale	Dissipative particle dynamics Lattice Boltzmann method Off-lattice MC method
Macroscale	Finite element method Finite difference method Free Galerkin method
Multiscale	Combination of methods

From the microscopic point of view, Tian et al. [97] used the non-equilibrium molecular dynamics (NEMD) simulation method. They described the impacts of different factors, such as particle orientation and arrangement, interface mismatch, interface density, and particle polydispersity. At the mesoscale level, the Lattice Boltzmann method was used by Zhou and Cheng to calculate *κ_c_* in two stages; the composite microstructure is first constructed using MC simulation followed by simulating the heat transport through the microstructure using LBM simulation [98]. Aligned fillers embedded in the polymer matrix found to be an effective way to improve *κ_c_* significantly. Using MD approach, Liu et al. studied the *κ* and the impacts of filler volume fraction for aligned CNT-PE PNC.

Furthermore, using EMA model, ITR was also calculated under the different conditions of the volume fraction of CNTs and interface defects. The results showed that *κ_c_* was better along the direction of CNTs; with increased volume fractions, *κ_c_* reduced with increased ITR as about −1 the power index [99]. In another work, the morphological and phonon characteristics of weaved PE and PNC of weaved PE-carbon nanotubes junctions (CNTJ) were analyzed using MD simulation. The Maxwell−Eucken model was also employed to predict the variation in the *κ_c_* with filler content. The MD results confirmed that the *κ*_c_ is 5.3-fold that of weaved PE, the while Maxwell−Eucken model showed that the intrinsic *κ_p_* plays a pivotal role in the *κ_c_* under low filler loadings [100].

Moreover, *κ* of PNC composed of cross-linked PE chains and functionalized single and double wall CNTs was calculated using reverse NEMD simulations. The simulations showed that with an increased weight percentage of the functional groups on CNTs walls, *κ_c_* of PNC was found to decrease. It was also observed that *κ_c_* was comparatively less sensitive to the increasing number of nanotube walls than the weight percentage of functional groups [101].

The ITR between polymer and filler can be treated as the temperature gap, and it dramatically influences the composite overall *κ* [102]. At the mesoscale level, using MD simulation, Bui et al. determined the ITR through phonon transmission probability using off-lattice Monte Carlo (MC) simulation and acoustic mismatch model. Similarly, Mortazavi and co-workers calculated the ITR of composites with different particles of different shapes using the macro-three-dimensional finite element method, and their results revealed that the ITR for non-spherical particles is less than that of spherical particles [103,104]. To discover the impact of orientation and dispersion evolution of CNTs *κ*_c_, Wang et al. [105] used dissipative particle dynamics (DPD) simulations under extensional shear coupled flow conditions. Furthermore, Sun et al. [106] studied the impact of the orientation of 2D filler such as h-BN on *κ*_eff_ of the polymer composites using FM modeling. The effect of *κ_p_, κ_f_*, the filler size, aspect ratio, and orientation, and the ITR on *κ_c_* was also studied. It was found that with increased filler loading, *κ_c_* increased as a power function; *κ_c_* increased almost proportionally with increasing *κ_p_; κ_c_* decreased with ITR in a negative power function form. *κ_c_* did not exhibit any change with *κ_f_* when the in-plane *κ_f_* was higher than 100 W/m·K and out-of-plane *κ_f_* was higher than 2 W/m·K and did not change with filler size with the same aspect ratio. However, with increased filler aspect ratio, in-plane *κ_c_* was increased, while out-of-plane *κ_c_* was found to be decreased.

**Figure 10.**
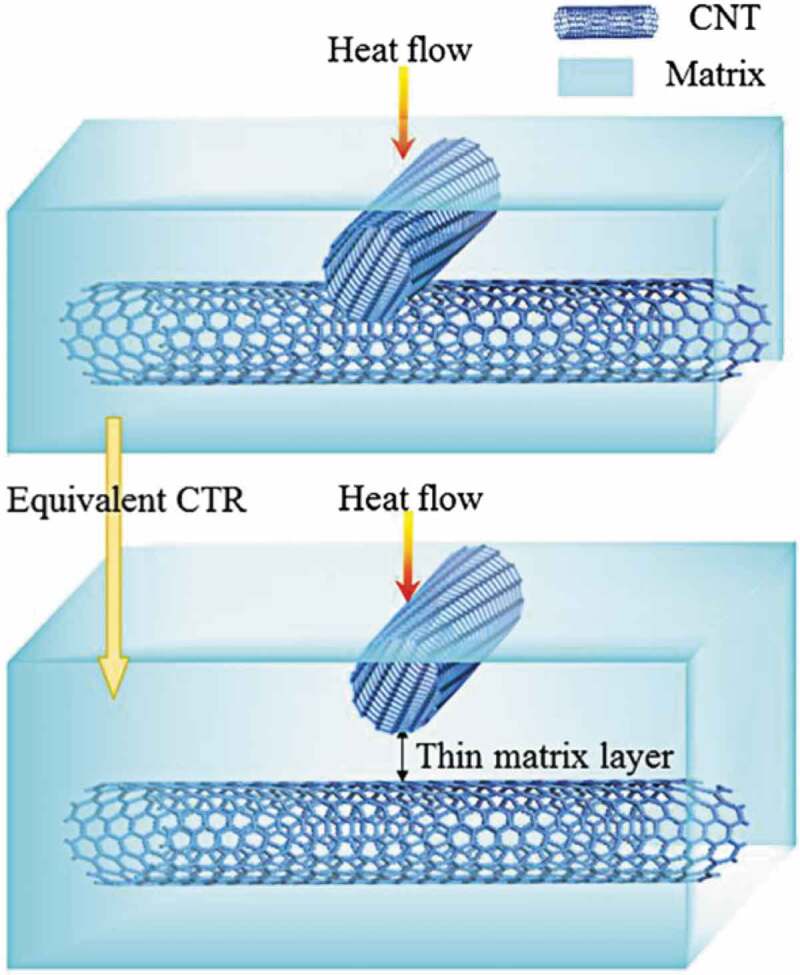
Schematic of the equivalent contact thermal resistance between the CNTs. ‘Reprinted from Ref [8]. Copyright (2018), with permission from Elsevier’

Among the macroscale models, Li et al. [107] developed a 3D computational model using a FE method to simulate the thermal behavior of randomly distributed single-walled carbon nanotube (SWCNT)/epoxy and SWCNT/polyolefin composites. They separately treated the phonons into three categories, i.e., phonons in SWCNT, matrix, and interface. They also treated the contact resistance between SWCNT as a thin matrix layer, as can be seen in Figure 10. They concluded that SWCNTs with larger diameter leads to higher *κ_c_*.

Multiscale models use different time and length scales via a combination of various methods to predict the *κ_c_*. In one of the reports, the authors calculated the ITR using a microscale model and predicted *κ*_c_ was using a mesoscale model. Moreover, the multiscale model predicted that graphene is more efficient in enhancing *κ_c_* compared to CNT because of the smaller Kapitza resistance and geometry of graphene [108]. In another simulation study, a multiscale method comprised of NEMD, pump-probe technique (PPT), and finite element (FE) method was used to study the impact of different 2D nano-sized fillers (graphene, C_3_N, and C_2_N) on *κ* of PE-based PNC. In this method, first, NEMD simulations were used to evaluate the *κ* of amorphous PE at the atomic scale, followed by the use of PPT to determine the interfacial thermal conductance (ITC) between filler and PE. Finally, using the results from MD simulations, FE-based 3D models of PNC were constructed to evaluate the *κ_eff_* at the microscale. The modeling results revealed that intrinsic *κ_f_* was the dominant factor in defining the *κ_eff_* of PNC, where the PNC containing graphene exhibited comparatively highest *κ*_eff_ in comparison with other fillers. However, ITC between all 2D fillers and PE plays a relatively less significant part in the heat transfer in PNC.

Furthermore, *κ_eff_* of PNC was found to decline with decreasing the aspect ratio of fillers at constant volume fractions (1.0%) [109]. Similarly, the effect of CNT functionalization on *κ_c_* of PE-based PNC was studied using a multiscale approach composed of large-scale MD, FEM simulations, and effective media modeling. By considering all the studies, the functionalization of CNTs causes a negative effect on the thermal transport in PNC containing randomly distributed CNTs due to the overwhelming effect of loss of intrinsic *κ_f_* and size of the thermal coupling zone [110].

## 4. Thermal conductivity of polyolefin composites

### 4.1 Carbon-based thermally enhanced polymer composites

Graphite-based materials belong to that class of conductive material, which have very high intrinsic *κ* with a relatively lightweight. Materials like graphite, exfoliated graphite, graphite nanoplatelets, carbon nanotubes, carbon fiber, conductive carbon black, and graphene have been extensively studied as conductive fillers to improve *κ_c_* [111]. Graphite and graphite-based fillers feature exceptional excellent properties such as extremely high strength and stiffness, high *κ* and abundant availability, and low cost (in some cases). These fillers have been incorporated into different polyolefin polymer to enhance their *κ*. Herein, we analyze the processing of polyolefin/graphite and graphite-based composites and their *κ*_c_.

#### 4.1.1 Graphite

Graphite is a naturally abundant layered carbon material. It consists of layers of carbons atoms hexagonally bound to each other by covalent bonds with an interatomic separation of 0.142 nm and an interlayer separation of 0.335 nm [112]. Graphite can be converted into different forms, such as expanded graphite (EG), graphite nanoparticle/exfoliated graphite (GNP), and graphite flakes (GF) to facilitate dispersion into the polymer matrix. EG is commonly synthesized using graphite intercalated compounds (GICs) exposed to sudden temperature increase for a short period. The sudden thermal shock of GICs results in light and worm-like structure whose thickness may vary from 100 to 400 nm. To produce GNP, EG can be further exfoliated using ultrasonication. GNP consists of small stacks of graphite layers that are 1 to 15 nanometers thick, with diameters ranging from sub-micron to 100 μm [99]. However, the production of GNPs is a costly, time-consuming, and not environmentally friendly process.

**Figure 11.**
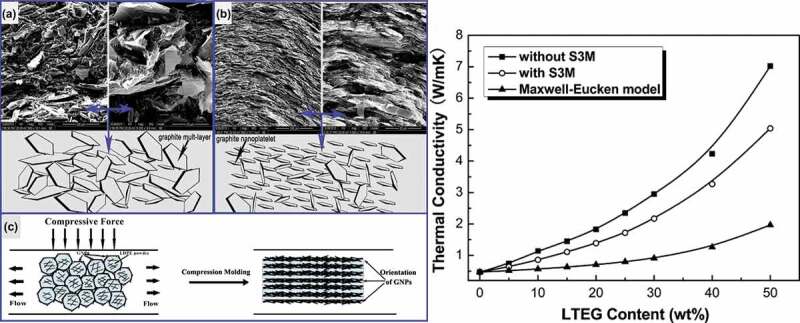
(L) Structural models based on the scanning electron microscopy (SEM) observations of LTEG/LDPE composites without (a) and with (b and c) S3M at 50 wt.% graphite loading showing the formation of conducting paths and orientation of GNPs. (R) The experimental thermal conductivity and theoretical thermal conductivity for LTEG/LDPE composites without and with S3M as a function of graphite content. ‘Reprinted from Ref [114]. Copyright (2013), with permission from Elsevier’

A very high *κ*_c_ of 12.4 W/m·K was reported for melt blended PP/EG composite containing 80 wt. % EG [113]. Wu et al. fabricated low-temperature expandable graphite (LTEG)/low-density polyethylene (LDPE) composites with high *κ_c_* via an in situ expansion melt blending process followed by solid-state shear milling (S3M) for 20 cycles to produce GNPs/LDPE composites (Figure 11) [114]. It can be seen in Figure 11 that milled composites exhibited lower *κ_c_* (5.1 W/m·K) compared to unmilled composite (7.02 W/m·K) at all filler loadings. This behavior was attributed to the difference in the formation mechanism of thermal conducting paths related to graphite filler having different morphologies and internal. Compared to GNPs, the macro-sized graphite formed a network throughout the polymer matrix due to partial aggregates of expanded graphite which lacking in case of treated samples [114]. In a similar report, LDPE/LTEG composite with high *κ_c_* of 11.28 W/m·K at 60 wt.% was processed by in situ expansion melt blending process. The very high *κ_c_* is attributed to the aggregation of graphite particles within the matrix and facilitates the improvement of *κ*_c_ [115].

**Figure 12.**
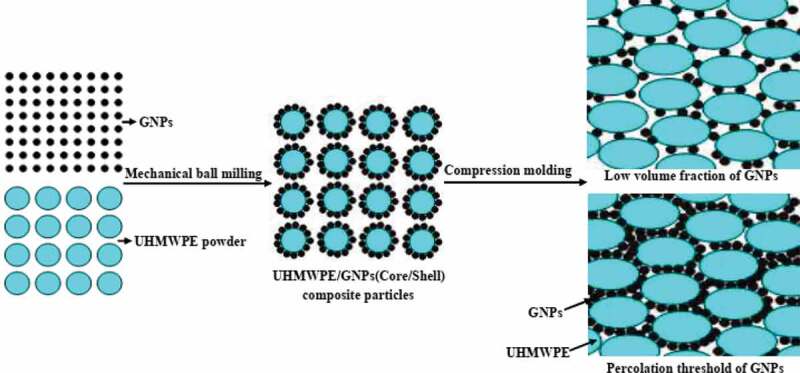
Schematic of thermal conductivity mechanism of the GNPs/UHMWPE nanocomposites reproduced from Ref [116] with permission from the royal society of chemistry

The development of an efficient connection between the particles of highly conductive fillers to reduce filler-filler thermal resistance plays a vital role in designing an efficient composite system. Gu et al. used ball milling to fabricate GNPs/UHMWPE PNC followed by hot-pressing. The PNC exhibited segregated structures with GNPs concentrated at the interface with UHMWPE after hot pressing, as shown in Figure 12. The fabricated PNC exhibited a high *κ*_c_ of 4.6 W/m·K at 40 wt.% owing to the formation of multidimensional thermally conductive networks between GNPs [116].

**Figure 13.**
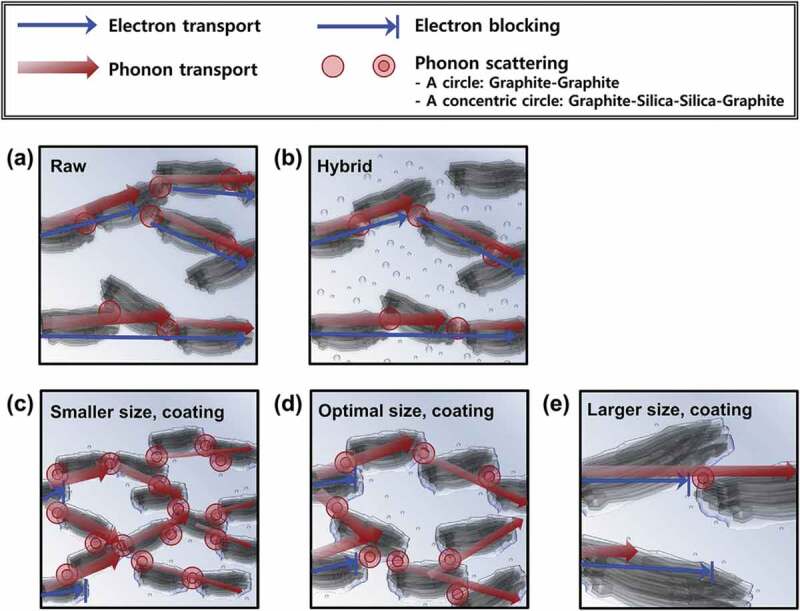
Electron-blocking effect and interfacial thermal resistance (ITR) in the composites in terms of filler types: (a) raw, (b) hybrid, and coated fillers with size of (c) 12, (d) 23, (e) 100 µm. ‘Reprinted from Ref [117]. Copyright (2018), with permission from Elsevier’

Using GNPs of different *a*, i.e., <100 and 1500 and different diameters (<1 µm and 15 µm), Kalaitzidou et al. [46] prepared PP composites using melt blending. At 10 vol.% GNP loading, *κ*_c_ was higher using high *a*, indicating reduction in the thermal contact resistance because of the large lateral dimension. Kim et al. [117] studied the use of silica-coated graphite and silica-graphite hybrid fillers to improve *κ*_c_ of HDPE composite. They proposed a scheme of phonon conduction paths and filler-filler interfacial resistance embedded in the polymer matrix, as shown in Figure 13. An optimal filler size is essential for improving *κ*_c_. Large particle size provides longer paths for the phonon conduction, but less coverage compared to a smaller size, whereas small size fillers have large interfacial area causing phonon scattering. Furthermore, composites using coated fillers were found to have superior *κ*_c_ compared to composites using uncoated graphite particle fillers. Table 3 summarizes the enhancement of *κ* of PE and PP using graphite-based materials.

**Table 3.** Thermal conductivities of polyolefin/graphite-based composites. AR stands for aspect ratio

No	Matrix	Filler	Loading (%)	*κ*_c, max_ (W/m·K)	Max *κ*_c_ enhancement (%)	Processingtechnique	Year [Ref.]
1.	PP	Exfoliated graphite nanoflakes (AR <100 and ~1500)	3–25 vol.	1.2	~413 (AR<100)	Melt blending	2007 [46]
2.	LDPE	Low-temperature expandable graphite (LTEG)	5.0–50 wt.	7.02	~1400	Melt blending followed by Solid-state shear milling	2013 [114]
3.	LDPE	Low-temperature expandable graphite (LTEG)	5.0–60 wt.	11.28	~2400	Melt blending	2013 [115]
4.	UHMWPE	GNP	0–21.4 vol.	4.62	900	Powdered ball milling	2015 [116]
5.	Recycled HDPE	Graphite flakes	1–20 wt.	1.31	~250	rotor milling	2016 [118]
6.	HDPE	silica-coated graphite	0–30 vol.	1.5	~440	Chemical modification and melt blending	2018 [117]
7.	PP	EG	10–80 wt.	12.4	~5400	Melt blending	2019 [113]

#### 4.1.2 Carbon nanotube

Carbon nanotubes (CNTs) are long cylindrical molecules consisting of hexagonal arrangement of sp^2^ hybridized carbon atoms, analogous to rolling up single or multiple sheets of graphene, i.e., single-walled carbon nanotubes (SWCNTs) or multiwalled carbon nanotubes (MWCNTs). The reported *κ* of about 3000 W/m·K and above 2000 W/m·K for MWCNTs and SWCNTs, respectively. The high *κ* and *a* of carbon nanotube (CNT) make it a good filler for the fabrication of thermally conductive PNC. Similar to other conductive fillers, the thermal conduction in CNTs also takes place via phonon conduction mechanism. *κ* of CNTs depends on several factors such as atomic arrangement (chirality), the tube diameter and length, the number of structural defects, as well as on the presence of impurities [12].

Molecular dynamics study on aligned PNC of CNT/PE indicated a very high *κ*_c_ could be achieved [119]. In another study, PNC of SWCNT with LDPE and HDPE was prepared by optimizing the crystallinity of polyethylene, crystalline alignment in the matrix, and aligned SWNT to improve *κ*_c_. The PNC prepared from isotropic PE with ∼78% crystalline phase showed higher *κ*_c_ of ~3.5 W/m·K at *φ* ∼0.2 twice than that of PE with ∼33% crystalline phase. This behavior can be attributed to reduced ITR resulted due to the large number of crystalline bridges between the nanotubes and the crystalline phase. Similarly, fibers were also produced from these PNC with low loadings, which resulted in materials with higher *κ* along the alignment direction [120]. The a, i.e., length to diameter ratio, is an important parameter that directly affects *κ*_c_. Evgin et al. investigated HDPE/CNTs nanocomposite using different *a* in the range of 200 to 3000. Compared to HDPE, at 10 vol.%, *κ*_c_ enhancement reached 63% (0.63 W/m·K) and 97% (0.76 W/m·K) for the nanocomposite with low and high *a* of CNT, respectively. This more significant enhancement for the high *a* CNT was attributed to the creation of a percolated network and enabling the travel of phonons over a longer distance without transitions from particle to particle [121]. Table 4 summarizes the enhancement *κ* in of PE and PP using CNTs-based material.

**Table 4.** Thermal conductivities of polyolefin/CNTs nanocomposites

No	Matrix	Filler	Loading (%)	*κ*_c, max_ (W/m·K)	Max. *κ* enhancement (%)	Processing technique	Year, Ref.
1.	LDPE	SWCNT	30 wt.	1.8	~700	hot-coagulation and fiber spinning	2007 [120]
2.	HDPE	SWCNT	30 wt.	3.5	~700	hot-coagulation and fiber spinning	2007 [120]
3.	PP	MWCNT	16 wt.	0.55	300	Melt blending	2014 [122]
4.	HDPE	MWCNT	10 vol.	0.75	~200	Melt blending	2016 [121]
5.	PP	Functionalized MWCNT	5 vol.	0.20	200	Melt blending	2016 [123]

#### 4.1.3 Graphene

Graphene sheets are one-atom-thick 2D layers of hexagonally ordered of sp^2^-bonded carbon. It exhibits a very high *κ_f_* of up to ~5000 W/m·K due to the covalent sp^2^ bonding between the carbon atoms. The Discovery of graphene has opened the gateway for novel functional polymer PNC with many superior properties compared to property increments usually achieved from other conventional fillers [124]. Graphene has a higher surface-to-volume ratio compared to carbon nanotubes (CNTs) as the inner surface of the nanotubes is not accessible to the polymer chains and lower cost making graphene more favorable than CNTs [125]. However, graphene exhibits anisotropic *κ*_c_ and shows extremely low through-plane *κ*_c_ due to weak van der Waals coupling [126]. Using molecular modeling Xie et al. confirmed that graphene nanosheets are more effective in *κ*_c_ enhancement of PNC than CNTs [127]. Recent studies showed that small interfacial thermal conductance (ITC) could play a crucial role in phonon transport across the filler and the matrix phases. It was reported that exceptionally low ITC ~12 MW/m^2^·K and 30 MW/m^2^·K in another case restricts the heat transport to a great extent in composite incorporated with highly conductive fillers [128,129]. Chemical functionalization of graphene is an effective route to decrease the interfacial resistance. However, other studies demonstrated that a lower grafting density could lead to a decrease in *κ_c_*. In contrast, with increasing grafting density, *κ*c reduction becomes slower and levels out at 80% enhancement. This behavior originates from the softening of high- and weaken the in-plane energy transfer. Wang et al. simulation study indicated that functionalization lowers *κ*_c_. However, a critical filler length was found, beyond which functionalization fails to enhance the overall *κ*_c_ [130]. Khanam et al. studied the effect of GNP diameter on *κ*_c_ and attributed the higher *κ_c_* at larger GNP diameter to agglomeration; it contributed to the formation of conductive pathways, while lower surface area reduced the scattering of phonons at interfacial defects [131]. Saeidijavash et al. [25] reported an increase of 60% in *κ*_c_ of the aligned PE-GNP composite using 10 wt.% GNP relative to oriented pure PE using mechanical strain. Table 5 summarizes the enhancement of *κ* in PE and PP using graphene-based materials.

**Table 5.** Thermal conductivities of polyolefin/graphene-based nanocomposites

No	Matrix	Filler type	Filler loading (%)	*κ*_c, max_ (W/m·K)	Max. *κ*_c_ enhancement (%)	Processing technique	Year [Ref.]
1.	PE	Functionalized Graphene	10 vol.	27.5 with 5 μm filler	6700	Simulation	2014 [130]
2.	LLDPE	GNP	10 wt.	0.5	~140	Melt blending	2016 [125]
3.	LLDPE	GNP	10 wt.	0.7	~200	Melt blending	2016 [131]
4.	PE	GNP	10 wt.	5.9	~1600	Melt bending and alignment	2017 [25]

### 4.2 Metallic fillers

The incorporation of metallic particles into the polymer matrix improves both *κ*_c_ and the composite electrical conductivity and reduces the dielectric breakdown voltage but increases the density significantly due to higher metal loadings. Micro- and nano-size particles of aluminum, silver, copper, zinc, bronze, and nickel used for *κ*_c_ improvement and the improvement of *κ_c_* depends on the *κ* of the metallic fillers, the particle shape and size, the volume fraction, and spatial arrangement in the polymer matrix [12,13]. Table 6 summarizes the enhancement of *κ*_c_ for PE and PP composites with metallic particles.

**Table 6.** Thermal conductivities of polyolefin/metal composites

No	Matrix	Filler type	Loading (%)	*κ_c_*, _max_ (W/m·K)	Max. *κ_c_* increase (%)	Processing technique	Year [Ref.]
1.	HDPE	bronze	24 vol.	1.85	~370	Roll Milling	2001 [132]
2.	HDPE	aluminum	33 vol.	3.6	~670	Powder Mixing + compression	2003 [134]
3.	PP	Cu (different size)	42 vol.	2.34	~940	Melt Mixing	2005 [135]
4.	LDPE, LLDPE	Cu	24 vol.	0.76 (LLDPE)	~220		2006 [136]
5.	LMDPE	Al	40 wt.	1.04	~260		2009 [137]
6.	HDPE	Cu	25 vol.	1.7	~380		2009 [133]
7.	LLDPE	Cu	25 vol.	1.6	~358		2009 [133]
8.	LDPE	Cu	25 vol.	1.5	~335		2009 [133]
9.	HDPE	Nano- Cu	5 vol.	0.56	~125		2009 [133]
10.	LLDPE	Nano- Cu	5 vol.	0.53	~118		2009 [133]
11.	LDPE	Nano- Cu	5 vol.	0.52	~116		2009 [133]
12.	HDPE	Ni	30 vol.	1.99	~425		2013 [138]
13.	PP	CuAg alloy	5 wt.	1.28	~600		2020 [139]

Neagu et al. studied the *κ*_c_ of HDPE composites containing powder of copper, iron, bronze, or zinc and prepared by roll milling. At filler concentrations above 16%, *κ*_c_ increased with the increase in the metal content due to the formation of agglomerates and conductive chains [132]. Molefi et al. compared the effect of particle size on *κ*_c_ of the micro- and nano-sized HDPE/Cu composites but found no difference *κ*_c_ for composites containing 5 vol. % nano-or micro-size copper. However, at 25 vol. % only micro-composite were prepared, which exhibited a *κ* of 1.7 W/m·K [133].

Furthermore, Krupa et al. [138] reported a *κ*_c_ of 1.99 W/m·K for melt mixed HDPE and nickel micropowder composites, achieved at a very high loading of 30 vol.% filler. The electrical conductivity percolation concentration of the filler was 8 vol.%. On the contrary, using silver-rich copper-silver alloy nanoparticles at a very low loading of 5 wt. %, a *κ_c_* of 1.28 W/m·K was achieved for melt mixed isotactic PP-based PNC. These results also indicate that it is not so simple to predict why one filler gives better *κ*_c_ than the other because many factors such as the filler shape, size, intrinsic *κ*_f_, affinity to the host matrix, dispersion, orientation, etc., must be taken into account while selecting filler to improve the *κ_p_* [139].

### 4.3 Ceramics filler

Ceramic-reinforced polymers have gained more attention in recent years due to their high *κ* and electrically insulating nature. Due to the lack of free electrons required for heat conduction in ceramic materials, the heat transfer predominantly occurs through phonons. In some cases, the strong interatomic bonding and crystal structure of some of the ceramic fillers such as BN, SiC also reduce the phonon scattering. However, some fillers, such as AlN particles, easily hydrolyze. Generally, *κ_c_* achieved using ceramic fillers depends on the filler packing density, particle size and size distribution, and surface treatment.

#### 4.3.1 Boron nitride-based composites

**Figure 14.**
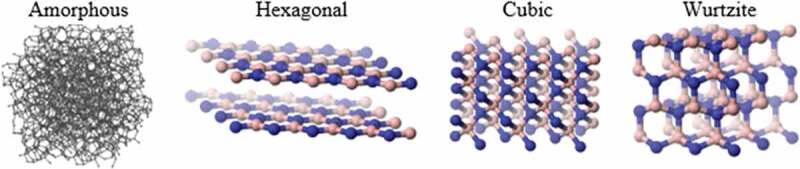
Different structures of boron nitride. Reprinted from Refs [140,144]

Boron nitride (BN) is a compound of boron and nitrogen with good lubricant and abrasive properties, high *κ*, and electrically insulating nature. Different arrangements of boron and nitrogen atoms give rise to other structures, including i) amorphous BN (a-BN), which is similar to amorphous carbon and lacks a long-distance arrangement of the atoms; ii) hexagonal BN (h-BN), which is a layered structure analogous to graphite; iii) cubic BN (c-BN), which is similar to diamond; and iv) wurtzite BN (w-BN), which is analogous to lonsdaleite. The rings between ‘layers’ form a boat configuration, as can be seen in Figure 14. Among different forms of BN, h-BN has recently attracted substantial attention due to its good heat dissipation ability.

The rare combination of its electrically insulating nature and high *κ_f_* makes h-BN a promising candidate to fabricate PNC as thermal management material compared to its graphite/graphene counterparts. The in-plane and through-plane thermal conductivities of h-BN are up to 400 W/m·K and approximately 3 W/m·K, respectively [140–142]. Like graphene, exfoliation of h-BN also produces two-dimensional (2D) material comprising single-layer or few-layers stacked together through weak van der Waals force. h-BN can be exfoliated using chemical, thermal, micromechanical cleavage, and sonication assisted processes producing the 2D h-BN nanosheets (hBNNs) [143].

##### 4.3.1.1 Hexagonal boron nitride

**Figure 15.**
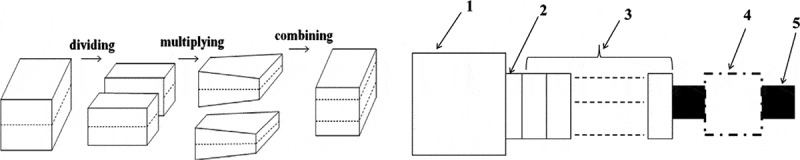
Schematics of (left) laminating-multiplying elements and (right) multistage stretching extrusion experimental system: 1-single screw extruder; 2-connector; 3- laminating-multiplying; 4-water cooling block; 5-sample. ‘Reprinted from Ref [146]. Copyright (2013), with permission from Elsevier’

Recently, BN-based fillers, specifically h-BN, have been reported to enhance *κ_c_* of PCs. To improve *κ_c_* of h-BN-based PCs, different approaches have been adopting, such as the use of large filler concentration, coupling agents, filler functionalization, and force field and processing to improve the dispersion. Unfortunately, the required large filler concentration deteriorates the composite mechanical properties and processability. Furthermore, filler compatibilization via functionalization and use of coupling agents are time, energy, and solvent consuming, and often reduces the high filler *κ_f_* leading to formation of inefficient composites [145]. Among the processing methods, melt processing is well developed, economical, and most widely applied method for thermoplastic polymers and their composites. However, in terms of quality of the filler dispersion, melt mixing ranks the lowest among other dispersion methods. Zhang et al. [146] introduced a novel technique based on melt processing called laminating-multiplying elements (LME) to improve the dispersion state of filler. As shown in Figure 15, melted polymers are divided and recombined using multistage stretching extrusion. In contrast, the flow behavior of the melt can be divided into three processes: dividing, stretching, and multiplying. *κ_c_* of PE/h-BN with 30 wt.% filler was increased from 0.99 to 1.21 W/m·K after 8 LME cycles indicating the improved dispersion state of the filler.

**Figure 16.**
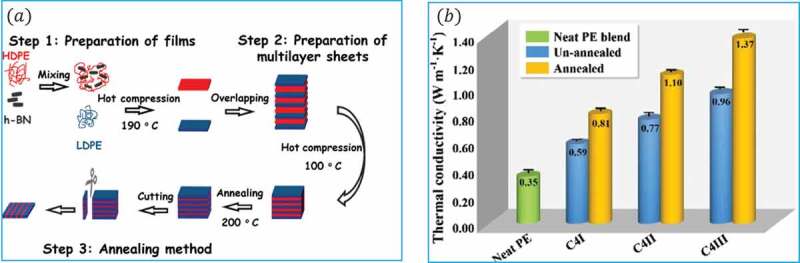
(a) Schematic representation of the processing procedure, (b) Thermal conductivity of annealed at 200°C, and unannealed samples for different thicknesses. ‘Reprinted from Ref [147]. Copyright (2018), with permission from Elsevier’

Similarly, Yang et al. [147] reported a new method to fabricate thermally conductive composites at low filler concentration by creating a multilayer structure. The composite was fabricated by alternating HDPE/h-BN and LDPE layers, followed by an annealing procedure (Figure 16). The annealing method was used to promote the percolation and orientation of fillers the composite layer via unidirectional interdiffusion of polymer molecules across the interface. This diffusion phenomenon can be attributed to the diffusion of HDPE chains from the composite layer to the neat LDPE driven by a compositional gradient. The through-plane *κ* of the composite was found to be increased from 0.49 W/m·K to 1.37 W/m·K (C4III) at 5.97 vol.% with the optimum initial layer thickness (10 µm), filler loading (5.97 vol.%), and annealing time (2 h) [147]. The influence of h-BN particle size and polymer melt viscosity on *κ* was also studied. Large h-BN size is more effective in increasing *κ* of PP/h-BN composite due to the formation of a network structure of large h-BN at a lower loading than small h-BN. Furthermore, *κ_c_* of PP/h-BN composite with high PP melt flow index (MFI) was slightly higher than that of PP with low MFI [142].

##### 4.3.1.2 Hexagonal boron nitride nanosheets

**Figure 17.**
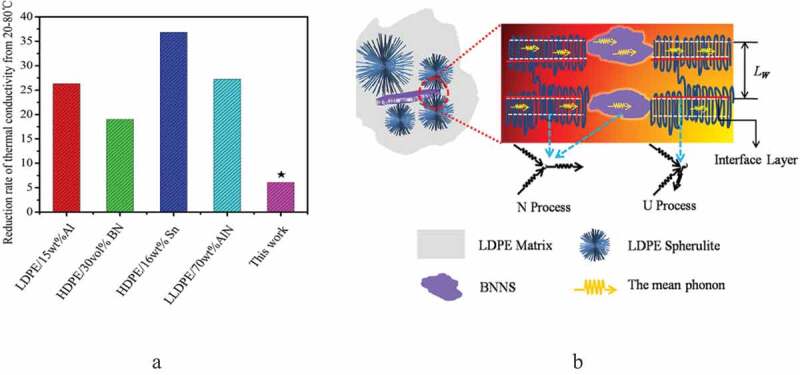
(a) Reduction in thermal conductivities of polyethylene-based composites at different temperatures, (b) the schematic of the bridging effect between BNNS and spherulites. ‘Reprinted from Ref [148]. Copyright (2019), with permission from Elsevier’

High *κ*_c_ usually requires high filler concentration, which could result in the deterioration of other polymer properties. Therefore, improvement of *κ_c_* at lower filler contents is always desired not only to avoid the negative impact of the high filler loading on other polymer properties but also to reduce the cost of the nanocomposite. Li et al. [148] fabricated nanocomposite of LDPE with hexagonal boron nitride nanosheets BNNs (<5 wt.%) and measured their *κ_c_* as a function of temperature (20–80°C). *κ_c_* of the composite containing 5 wt.% BNNs was increased by 22% at room temperature and 48% at 80°C relative to *κ* of LDPE. Figure 17 shows *κ_c_* reduction versus temperature (20°C to 80°C) of PE-based composites highly filled with comparatively large-size filler. Compared to BNNS nanocomposite, a high rate of *κ*_c_ reduction was observed in composites containing large-size filler. The improved *κ*_c_ of LDPE/BNNs nanocomposite at high temperature was mainly attributed to the bridging effect between BNNs and LDPE spherulites. In situ SAXS studies revealed that at high-temperature, BNNs were preventing the LDPE lamellae structure from thermally expanding and maintaining lamellae number to a higher level in the composites resulting in more phonon pathways [148]. Also, compared to micro h-BN particles, nanopowder resulted in good mechanical properties along with *κ_c_* values significantly lower than for micro-sized fillers [145]. Table 7 summarizes the reported enhancement in κc of polyolefin composites with hBN filler systems.

#### 4.3.2 Other ceramic fillers

**Figure 18.**
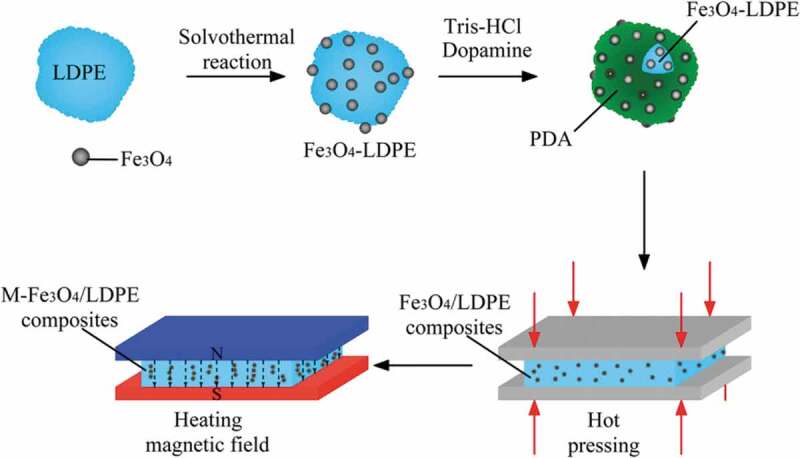
Schematic illustration of the preparation of the Fe_3_O_4_/LDPE and M- Fe_3_O_4_/LDPE composites ‘Reproduced from Ref [150]. Copyright (2019), with permission from Springer Nature’

Other ceramic fillers based on metal oxides and metal carbides were also used to fabricate thermally enhanced polyolefin composites. Chi et al. [150] used external electric and magnetic fields to improve the distribution of Fe_3_O_4_ in LDPE composites prepared and coated by solvothermal reaction (Figure 18). Compared with the Fe_3_O_4_/LDPE composites, the magnetically aligned Fe_3_O_4_/LDPE composites exhibited higher *κ_c_* at 7.0 vol.% Fe_3_O_4_ loading. SEM of the composites revealed that the Fe_3_O_4_ nanoparticles were distributed in the LDPE matrix without agglomeration and any evidence of defects and voids in the composite due to the solvothermal reaction process reducing the probability of Fe_3_O_4_ nanoparticles agglomeration. Morphology of the nanocomposite also showed that some Fe_3_O_4_ particles came in contact with each other and became short chains under the action of the magnetic field, resulting in the formation of efficient thermal pathways. It was found that the thermal enhancement of the magnetically aligned Fe_3_O_4_/LDPE composites reaching 46.27% at 7.0 vol.% compared to unaligned Fe_3_O_4_/LDPE composites, which showed *κ* improvement of 21.9% [150].

The high filler loading (>40 wt.%) usually causes severe problems, such as low fluidity and poor processability of the composites. Many studies focused on improving the rheological properties of the composites by introducing selective localization of the fillers in continuous regions using immiscible polymer within the host matrix. Rheological analysis of HDPE composites prepared using alumina whisker with different ratios of HDPE/PA6 immiscible blends showed at a ratio of 40/60 HDPE/PA6, liquid to solid behavior change was not observed with filler loading up to 40 wt.%. SEM images of the composites revealed sea-island morphology for 60/40 and 50/50 HDPE/PA6 ratios. The highest *κ_c_* was observed in the case of 50/50 HDPE/PA6 composites at 50 wt.% of filler because the higher effective filler concentration present in the PE phase compared to the other composites at the same filler concentration [151].

**Figure 19.**
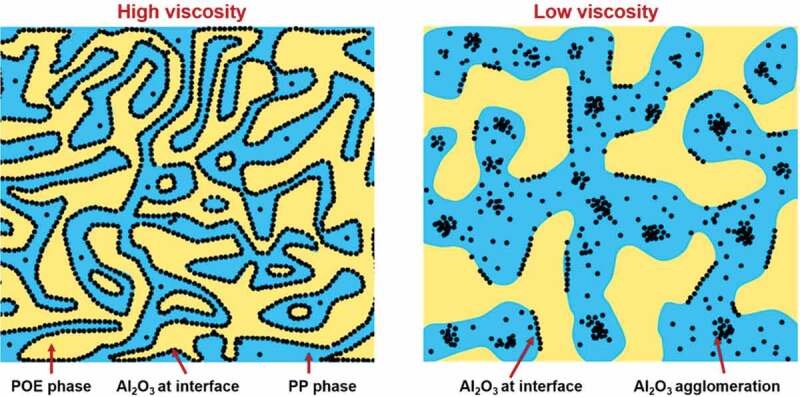
Schematic illustration of the morphology of PP/POE/Al_2_O_3_-RGT nanocomposites. The blue domains represent the PP phase, the yellow domains the POE phase, and the black circles Al_2_O_3_ nanoparticles. ‘Reprinted from Ref [152]. Copyright (2019), with permission from Elsevier’

A similar approach to localization of nanoparticles at the interface was adopted using blends of PP/polyolefin elastomer and their composite with Al_2_O_3_. Zhang et al. [152] investigated the effect of the selective localization at the interface and the phase domain size on *κ* the PNC. The Al_2_O_3_ nanoparticles and PP nanocomposite were first prepared using a reactor granule technique followed by melt mixing with polyolefin elastomer (POE). The Al_2_O_3_ nanoparticles thermodynamically preferred the localization at the interface between PP and POE (Figure 19). However, their study also showed that direct melt mixing of nanoparticles with blend generated severe agglomeration, which hindered the migration to the interface. The selective localization of Al_2_O_3_ nanoparticles at the interface was creating thermal conductive networks along with the co-continuous structure and eventually giving high *κ* [152].

**Figure 20.**
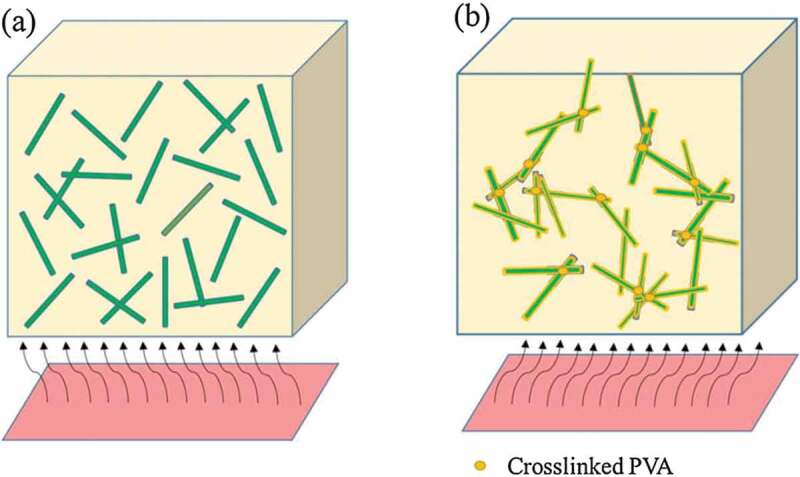
Thermal conductivity mechanism of HDPE/SCW(a) and HDPE/modified silicon carbide whiskers (b) composites. ‘Reprinted from Ref [153]. Copyright (2018), with permission from Elsevier’

Surface modification of fillers can improve their dispersion in the host matrix resulting in improved *κ*_c_. HDPE composites prepared using silicon carbide whiskers, unmodified or modified with cross-linked poly (vinyl alcohol), exhibited an increase in *κ*_c_ of 250% and 300% at 40 wt.% filler content, respectively. The higher *κ*_c_ enhancement is due to the formation of interconnection between the modified filler in the HDPE matrix (Figure 20) [153]. Table 8 summarizes the reported enhancement in *κ*_c_ of polyolefin composites with ceramic (others) filler systems.

**Table 7.** Thermal conductivities of polyolefin/hBN composites and nanocomposites

No	Matrix	Filler type	Filler loading (%)	κ_c, max_ (W/m·K)	Max. κ_c_ enhancement (%)	Processing technique	Year [Ref.]
1.	HDPE	h-BN	30 wt.	1.21	~ 300	Multistage stretching melt Blending	2013 [146]
2.	High & Low MFI-PP	h-BN (different particle size)	30 vol.	2 (Higher MFI-PP, large BN	~1000	Melt Blending	2013 [142]
3.	PP	Silane modified-BN	33.7 wt.	0.469	~240	Melt Blending	2015 [149]
4.	HDPE	h-BN sheets	5.97 vol.	1.37	~390	Melt blending and layer by layer assembly	2018 [147]
5.	HDPE	modified h-BN (nano, micro)	50 wt.	2.08 (micro h-BN)	~590	Roll milling	2017 [145]
6.	LDPE	BNNS	5 wt.	0.33	~123	LDPE Pulverizing & Melt mixing	2019 [148]

**Table 8.** Thermal conductivities of polyolefin/ceramic or (others) filler-based composites. PVA stands for poly (vinyl alcohol)

No	Matrix	Filler	Loading (%)	*κ*_c, max_ (W/m·K)	*κ*_c_ increase (%)	Processing technique	Year [Ref.]
1.	HDPE	Different particle size Al_2_O_3_	50 vol	0.56	~230	Roll Milling	2011 [154]
2.	LLDPE	ZnO	20 vol.	1.56	~380	Melt Mixing	2013 [155]
3.	PP	Hollow glass beads	20 vol.	0.16	~80	Melt mixing	2014 [156]
4.	LDPE	Fe_3_O_4_	1.7 vol.	0.384	~118	Solvothermal reaction	2017 [150]
5.	HDPE/PA6 50/50	Al_2_O_3_ whisker	50 wt.	0.96	~266	Melt mixing	2018 [151]
6.	HDPE	SiC/PVA	40 wt.	1.69	~410	Melt mixing	2018 [153]
7.	PP	Al_2_O_3_	20 wt.	0.74	~350	Melt mixing	2018 [157]
8.	PP/POE	Al_2_O_3_	11.1 wt.	0.44	~214	Reactor granule technology & Melt mixing	2019 [152]

### 4.4 Hybrid fillers

Hybrid fillers include a combination of fillers having a different shape, size, aspect ratio, and type to provide a synergistic effect and a balance in final composite properties. Hybrid systems usually provide a better thermally conductive network by forming bridges between fillers. A hybrid-filler system may also reduce the overall filler loading resulting in reduced composite viscosity. Fiber-reinforced PC exhibit excellent specific strength properties, e.g., strength/weight and/or stiffness/weight ratios. However, the anisotropic dependence of *κ* in fiber-reinforced plastic can cause difficult to dissipate heat. Using a hybrid of sisal/glass fiber, *κ_c_* of the LDPE-hybrid fiber composites was higher than the individual sisal fiber-reinforced composite due to the isotropic nature of the glass fiber [158].

**Figure 21.**
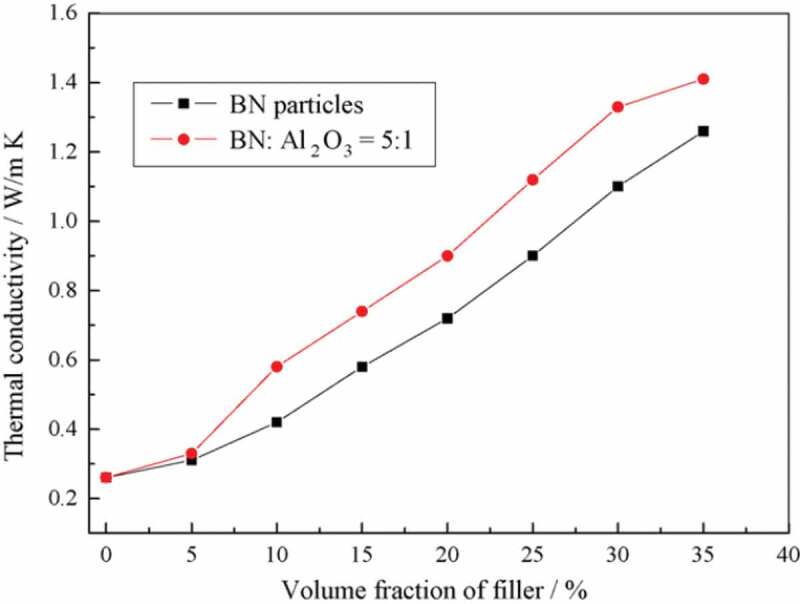
Thermal conductivity of HDPE composite containing hBN or hybrid of BN particles and short alumina fibers. ‘Reprinted from Ref [71]. Copyright (2007), with permission from Elsevier’

Similarly, a combination of BN particles with short alumina fibers also exhibited a higher *κ_c_* of HDPE composites compared to when only BN particles were used. Zhou et al. [71] prepared composites of HDPE with a hybrid of BN/Al_2_O_3_ short fiber (15 mm diameter and 1–2 mm length) using powder mixing. The combination of BN particles and short alumina fibers at a mass ratio of 5:1 resulted in higher *κ_c_* compared to using BN particles alone, as shown in Figure 21. The higher *κ_c_* achieved with the hybrid filler is due to the efficient phonon transfer through the formation of random conductive leading to the abundance of conductive paths.

**Figure 22.**
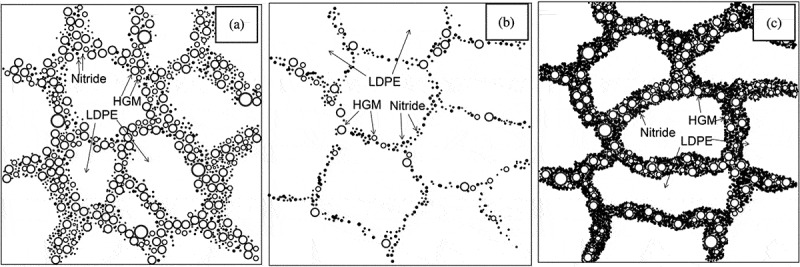
Schematic microstructure of composites prepared with different parameters: (a) volume ratio of nitride particles to HGMs is lower; (b) total filler volume fraction of filler is lower; (c) both volume ratio of nitride particle to HGM and total filler volume fraction of filler are high enough. ‘Reprinted from Ref [159]. Copyright (2014), with permission from Elsevier’

Zhu et al. prepared LDPE composite with hollow glass microspheres (HGM), BN, AlN particles for electronic packaging, and the optimal volume ratio of nitride particles to HGMs was 1:1. Figure 22 depicts the evolution of the microstructure of the composite incorporated with different amounts of hybrid fillers. It can be seen that at a low volume ratio of nitride particles to HGMs, the conductive network of nitride particles could not form effectively. At a higher volume ratio, the filler cannot fully interface with the LDPE matrix; thus, the thermally conductive network also could not form effectively form. However, when both the total volume fraction and ratio were high enough, most of the adjacent nitride particles were in contact and developed thermally conductive pathways. The heat flow can bypass the HGMs, which have low *κ*, and composites showed high *κ* [159].

**Figure 23.**
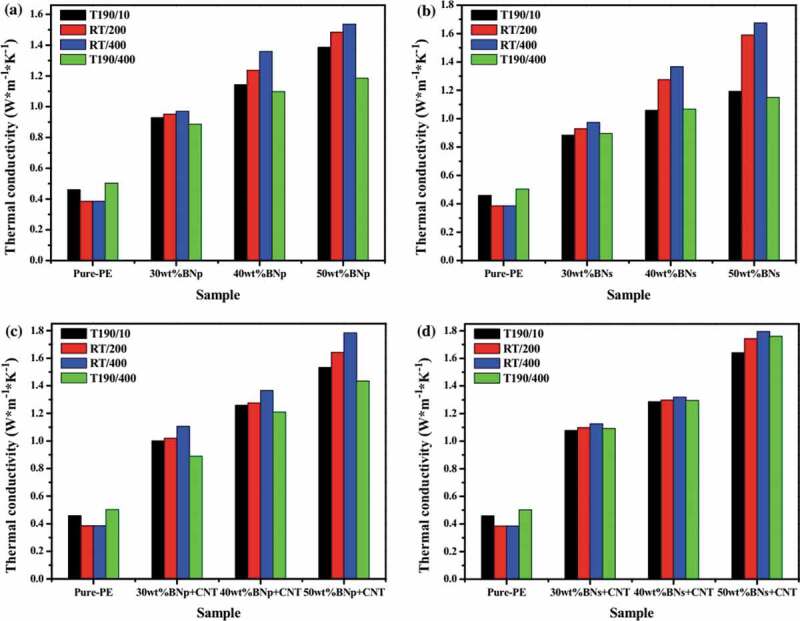
Thermal conductivities of UHMWPE and hybrid (BNs+CNTs) composites under various conditions. ‘Reprinted from Ref [160]. Copyright (2016), with permission from Elsevier’

The ITR of the PC can also be improved by constructing micro-nano-architectonics of the interface. The micro-nano-architectonic of filler-polymer and filler–filler interface mainly depends upon many factors such as dispersion and alignment of fillers, morphology, and contact area of filler/filler or filler/polymer interface and manufacturing/processing process [54]. The structure and shape of composites can be constructed during the manufacturing process. Ren et al. studied the influence of compression molding conditions on *κ* of UHMWPE-BN and UHMWPE-(BN + MWCNT) hybrid composites. The physical mixing of polymer and fillers was done using a high-speed mixer followed by compressing to prepare four different samples controlled by the preparation conditions. The first two samples were obtained by cold-pressing at room temperature under 200 MPa and 400 MPa followed by sintering at 190°C under 0.1 MPa for 5 min, designated as RT/200 and RT/400, respectively. The third and fourth samples were obtained by hot-pressing at 190°C under 10 MPa and 400 MPa for 5 min, designated as T190/10 and T190/400, respectively. The resulted composite morphology exhibited a segregated structure. The study showed that the compression molding technique has a significant influence on *κ_c_*, as indicated by high *κ* observed for all composites treated with the cold-pressing sintering process (Figure 23). This effect of the compression molding on *κ_c_* is due to the formation of an integrated filler network/segregated structure at RT molding, which was destroyed at high temperatures. However, the dispersion of fillers in the polymer matrix was also improved under high pressure and high temperature owing to the improvement in the polymer fluidity [160]. Others used combinations of micro-fillers and nano-fillers to create 3D thermally conductive network within the polymer matrix such that the nano-fillers bridges the micro-filler particles to create a 3D network. For example, using GNP with composites of isotactic polypropylene and h-BN, a 3D network was formed via the bridging effect of GNP between the h-BN particles. Similarly, a thermally conductive network was created in PP/CF composites by adding CNTs via coagulation precipitation technique. In these composites, CNTs acted as a bridge among oriented CFs [54,161,162].

**Figure 24.**
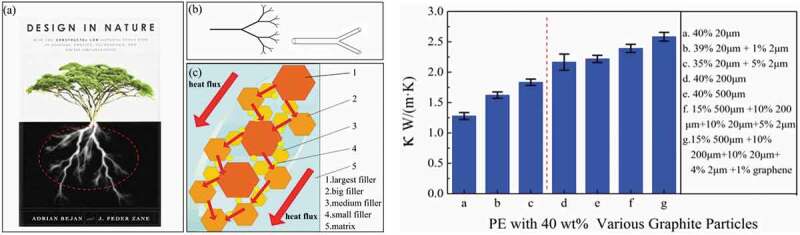
(a) A fractal root system of the tree; (b) a classical constructal network; and (c) diagram of several-sized fillers filling in the matrix, and c) thermal conductivities of HDPE with 40 wt.% various filling particles (the size unit is µm). Reproduced from Ref [164]

The constructal-theory network was used to design conducting paths to cool a heat-generating volume, inspired by the fractal root system of tree-shaped flows. The constructal-theory deals with designing conducting pathways with improved access (minimum flow resistance) (Figure 24a). In this theory, the modes of thermal transport with the highest and lower resistivity are placed at the small and large construct, respectively [163]. Liu et al. [164] adopted the same strategy and fabricated HDPE composites containing graphite-based fillers of different particle sizes. In these composites, large fillers were acting like backbones, whereas the smaller fillers represented the branches (Figure 24(b-c)). The highest *κ* about 2.51 W/m*·*K was found by incorporating a combination of 15 wt. % 500 µm, 10 wt. % 200 µm, 10 wt. % 20 µm, 4 wt. %, and 1.0 wt.% graphene (total filler 40 wt. %).

Kucukdogan et al. used red mud, which is an industrial waste, as thermally conductive filler for PP. Red mud mainly consists of a mixture of oxide of different metals such as iron, aluminum, silicon, titanium, and calcium with *κ_f_* of 11.7 W/(m·K). The composites with 50% volume fraction exhibited *κ*_c_ of 1.4 W/(m·K), which was almost 5 times higher than pure PP, showing potential reuse of waste in suitable applications [165]. Table 9 summarizes the reported enhancement in *κ* of polyolefin using hybrid fillers.

**Table 9.** Thermal conductivities of polyolefin and hybrid-filler-based composites

No	Matrix	Filler	Loading (%)	*κ_c_*, _max_ (W/m·K)	Max. *κ_c_* increase (%)	Processing technique	Year [Ref.]
1.	LDPE	sisal, glass, Hybrid	20 vol.	0.49 Glass fiber (ǁ@86°C)	~180	Solution mixing	2000 [158]
2.	HDPE	hBN-Al_2_O_3_ Short fiber	40 vol.	1.4 (Al_2_O_3_: BN = 1:5)	~500	Powder and melt mixing	2007 [71]
3.	PP	h-BN, AlN, MWCNT	70 wt.	1.2	~530	Melt blending	2014 [166]
4.	LDPE	hybrid hollow glass microspheres, AlN, BN	50 vol.	2.25 (AlN+HGM)	~720	Powder mixing and hot press	2015 [159]
5.	PP	Carbon fiber, CNT	40 wt.	1.23 (Axial)	~550	coagulation precipitation technique	2015 [162]
6.	UHMWPE	h-BN particles, h-BN sheet + MWCNT	50 wt.	1.794 (49 wt.%+ 1 wt.%, RT/400 MPa)	~390	Powder mixing and different compression	2016 [160]
7.	PP	h-BN, GNP	30 wt.-5 wt. GNP	0.73	~330	Melt mixing	2016 [161]
8.	PP-	Al_2_O_3_ + CNT-carbon black	15 wt.	0.68	~340	Melt blending	2016 [167]
9.	PE	CNT/Graphene	0.5 Gra-4.5 CNT vol.	1.3	~370	Melt blending	2017 [168]
10.	LDPE	epoxy & HGM, BN, Hollow glass microsphere	50 vol.	0.7 (30 vol.% BN and HGM, LDPE/Epoxy = 3:7	~370	Physical Mixing	2017 [169]
11.	HDPE	BN-CNT	30 wt.	2.671 (25BN,3CNT)	~600	Melt blending	2018 [170]
12.	HDPE	Graphite. graphene, CNT	40 wt.	2.51	~730	Powder Mixing and Melt-Extruding	2018 [164]
13.	PP	Red mud	50 vol.	1.4	~470	Melt mixing	2018 [165]
14.	LDPE	MWCNT/GNP	CNT/GNP = 5 wt.	0.42	~120	Melt blending	2018 [171]

## 5. Challenges and prospective directions

Although much progress is made during the past two decades in developing thermally conductive polyolefin composites, there are still several challenges to be addressed that require research efforts in the following aspects:

1. *κ_c_* is strongly dependent on the interfacial resistance between the polyolefin matrix and the filler. Therefore, a holistic strategy to improve such interface during the composite processing should be developed to enhance *κ*_c_ significantly.
2. The adverse impact of the high filler loading required to reach high *κ_c_* on the composite processability can be mitigated by, for example, the use of plasticizers. However, the impact of plasticizers or other means of mitigation on the composite conductivity and mechanical properties should be examined.
3. Using nano/exfoliated fillers may also reduce the required filler loading. However, due to their large surface area, nanofillers introduce a vast number of interfaces and may not improve *κ_c_* significantly.
4. The filler functionalization can improve the dispersion and interfacial resistance between polyolefin and the filler. However, functionalization of the fillers may contribute to phonon scattering resulting in lower *κ_c_*.
5. High *κ_c_* can be achieved at lower filler loading by considering and optimizing key factors such as filler dispersion, optimized filler size, filler hybridization, the shape of the filler, optimized number of present interfaces between filler and polymer, filler alignment and orientation, processing technique, polyolefin type, grade, etc.
6. Combining modeling with experimental inputs can play a key role in reducing the number of experiments. Furthermore, it can also provide insights on the dependence of *κ_c_* on *κ* of the matrix and the filler, the filler volume fraction, and the filler shape and morphology.
7. An improved *κ* of PE can be obtained by using a combination of conductive fillers and controlling PE microstructure via post-processing techniques such as drawing to orient the PE chains in the heat transfer direction. For instance, at relatively low fillers loadings, simultaneous alignment of PE lamellae and incorporated anisotropic fillers lead to substantial enhancement in *κ_c_* of PE composites [22].
8. Finally, the cost of thermally conductive composites and nanocomposites should be considered when a high loading of costly fillers are used. The development of such composites may eradicate the low-price advantage of polyolefin.

## 6. Conclusions

This review article reports the research progress on thermally conductive polyolefin composites and nanocomposites. The thermal conductivity of polyolefins can be enhanced by the incorporation of fillers with high intrinsic thermal conductivity. Although filled polyolefin composites exhibit improved thermal conductivity, high filler concentration also affects the polymer melt viscosity and hence affects the processability, mechanical stability, and product performance. Furthermore, the thermal conductivity of composites also depends on many factors such as the filler size, shape, *a*, loading level, dispersion, morphology, functionalization/interfacial compatibility, filler/polymer and filler/particle interfaces were also discussed in the review. The presence of functional groups on the surface of inorganic fillers improves the dispersion/distribution of the fillers and phonon transport across the interface; however, beyond a certain functionalization level, the surface functionalization could increase phonon scattering. The orientation of fillers with anisotropic *κ* plays a major role in determining *κ_c_*. The shape and size of filler are also crucial for *κ_c_* because large fillers and longer dimensions can have less filler to polymer interface, and consequently lower thermal interfacial resistance and vice versa for nanofillers. However, the optimized filler size would require obtaining an optimized number of polymer/filler interfaces to obtain suitable *κ_c_*. The use of hybridization fillers that combines fillers of a different type, shape, or size also help to obtain high *κ_c_* at lower loading. The use of hybrid filler can also be used to control the arrangement of the fillers. Furthermore, the blending of polyolefin with different polymers can generate a phase-separated polymer blend with an island-sea morphology containing double percolated regions, and consequently leading to higher *κ*_c_ at low filler loading.

Incorporating highly conductive fillers into polyolefin matrices remains the most promising approach to develop thermally enhanced polyolefin materials. However, significant advances are still required to obtain efficient thermally conductive composites in terms of high composite thermal conductivity at reasonable filler loading while maintaining or improving the mechanical properties and the processability necessary to meet the requirements of commercial applications.
